# Comparison of risks and costs of undiagnosed and diagnosed chronic kidney disease patients after the initiation of kidney replacement therapy in subsequent years. A Hungarian nationwide study

**DOI:** 10.3389/fmed.2026.1759417

**Published:** 2026-07-21

**Authors:** Levente Kovács, Zsolt Abonyi-Tóth, György Rokszin, Erzsébet Ladányi, Zsolt Fábián, Imre Boncz, István Wittmann, Boglárka Laczy

**Affiliations:** 1 Second Department of Medicine and Nephrology-Diabetes Center, Medical School, University of Pécs, Pécs, Hungary; 2 Department of Biostatistics, University of Veterinary Medicine, Budapest, Hungary; 3 RxTarget Ltd, Szolnok, Hungary; 4 National Dialysis Center, Miskolc Nephrology Center, Miskolc, Hungary; 5 Boehringer Ingelheim, Budapest, Hungary; 6 Institute for Health Insurance, Faculty of Health Sciences, University of Pécs, Pécs, Hungary

**Keywords:** chronic kidney disease (CKD), cost, end-stage kidney disease (ESKD), kidney replacement therapy (KRT), mortality, real-world data, nationwide, undiagnosed

## Abstract

**Introduction:**

Despite high prevalence, chronic kidney disease (CKD) often remains undiagnosed and untreated, leading to an increased risk of end-stage kidney disease (ESKD), kidney replacement therapy (KRT), comorbidities, and premature death. In this retrospective nationwide study, we examined whether a lack of CKD diagnosis is associated with adverse post-KRT outcomes among newly dialyzed patients with chronic ESKD in Hungary.

**Methods:**

Two cohorts—undiagnosed (CKD naive) and diagnosed (CKD non-naive) patients (aged > 18 years)—were compared. Cohorts were defined based on pre-dialysis (>2 months) diagnosis-coding and erythropoietin therapy. We compared the risk of primary endpoints after KRT initiation (including all-cause mortality, acute myocardial infarction, stroke, hospitalization for heart failure (HHF), lower limb amputation (LLA), cancer, kidney transplantation) using propensity score subclassification for 56 confounders (matched data). We also examined exploratory endpoints (total healthcare costs, key comorbidities, laboratory testing, medications) over 5 years pre-KRT and/or post-KRT (unmatched data). Data were collected from the National Health Insurance Fund registry.

**Results:**

We identified 38,013 ESKD patients on KRT in 2009–2023, of whom 10,810 started dialysis in 2014–2018. Among these, 33% were undiagnosed (CKD naive). This group experienced more adverse events as early as 3 months post-KRT, including higher deaths (27% vs. 7.8%), HHF (18.8% vs. 11.1%), and cancers (11.2% vs. 2.1%). They also had fewer kidney transplantations (4.6% vs. 14%) at 72 months than the CKD non-naive group. Median survival was markedly shorter for CKD naive patients (3 months) than for CKD non-naive patients (34 months). Cox regression revealed higher risks for CKD naive patients for all-cause mortality [HR: 3.66 (CI: 3.12–4.29), *p* < 0.0001], cancers [HR: 3.31 (CI: 2.55–4.29), *p* < 0.0001], HHF [HR: 1.81 (CI: 1.55–2.11), *p* < 0.0001], stroke [HR: 2.27 (CI: 1.08–4.76), *p* = 0.0307], and LLA [HR: 2.17 (CI: 1.17–4.04), *p* = 0.0146] at 1 month post-KRT. Total costs rose 7.5-fold in undiagnosed CKD patients compared to a 4.5-fold increase in diagnosed CKD patients over 5 years pre-KRT, driven by higher hospitalization spending. Drug therapy costs were consistently higher in diagnosed vs. undiagnosed CKD patients pre−/post-KRT.

**Discussion:**

Undiagnosed CKD patients experience significantly worse outcomes post-KRT and incur higher long-term costs related to inpatient care. These findings underscore the need for early CKD diagnosis and timely interventions to improve survival and optimize healthcare resource utilization.

## 1 Introduction

Chronic kidney disease (CKD) poses a significant public health problem, affecting over 850 million people worldwide (1). The clinical impact of CKD is related to multiple complications at the patient level, leading to increased morbidity and premature death (2). The economic burden attributed to the management of CKD, including conservative treatments and kidney replacement therapy (KRT), also imposes substantial costs on healthcare systems (3–6).

Global prevalence of CKD was estimated at 9.1% in 2017 (7). The updated overall prevalence of CKD was 13.0% for 2014–2022 in a recent meta-analysis (8). Drivers for the increasing prevalence of CKD include the epidemic of main risk factors, such as diabetes, hypertension, and the aging of the general population (1, 2, 7, 8). In alignment with global data, the number of CKD patients was projected to increase in Hungary, reaching 15.8% of the adult population by 2027 (9, 10). Of the projected CKD population, most patients (73–80%) are expected to remain undiagnosed, having higher mortality than those with a formal diagnosis (9, 10).

As CKD progresses, patients face an increasing risk of developing end-stage kidney disease (ESKD), while also being at higher risk for cardiovascular diseases (CVDs), which are the leading cause of mortality and often compete with the risk of needing KRT (11–14). Cancer-related deaths also contribute to the excess mortality seen in CKD due to increased cancer incidence (15). Patients with advanced CKD are more likely to develop coronary artery disease (CAD), stroke, and lower extremity artery disease (LEAD), leading to severe outcomes including heart failure (HF), acute myocardial infarction (AMI), and lower limb amputations (LLA), along with increased hospitalizations (6, 11, 14)—ultimately resulting in a higher burden of comorbidities and greater healthcare resource utilization.

Despite the high prevalence and mortality risk, the majority of CKD patients are undiagnosed and thus more likely to be untreated (6, 16–18). The REVEAL study showed that 61.6–95.5% of stage 3 CKD patients, when symptoms may be noticeable, were unreported (18). A delayed diagnosis by 1 year increased the risk for progression to stage 4–5 by 40%, KRT by 63%, and the composite of AMI, stroke, and hospitalization for HF (HHF) by 8% (19). Conversely, establishing the diagnosis promoted guideline-based care and clinical monitoring of patients, and mitigated the decline in kidney function (19).

CKD is the leading cause of health expenditures worldwide. Direct costs of diagnosed CKD and KRT were predicted to increase by 9.3% across 31 countries between 2022 and 2027 (3). Expenditures increase with progression from early stages to the transition to ESKD, and disproportionately more upon KRT initiation (3, 4). The proportion of CKD patients on KRT is very low (about 0.15%) (7), although 2–4% of the annual healthcare budget is funded for KRT in high-income countries (5). In Hungary, the treatment cost of diagnosed CKD is estimated to take 5.4% of the healthcare budget by 2027, most of which comprises kidney transplants (1.7%) and hemodialysis (1.5%) (10). Estimated expenses for CKD-associated AMI, stroke, and HF remain unchanged (10). Datasets of undiagnosed CKD were not used in these models (3, 10).

Controlled data assessing the long-term impacts of undiagnosed CKD on post-KRT morbidity and mortality are limited. We proposed that diagnosed patients with late CKD under nephrological care—defined as having a formal diagnosis and pre-dialysis erythropoietin (P-EPO) therapy—experience increased survival, reduced morbidity risks, and lower associated costs compared to undiagnosed CKD patients.

The objective of the present nationwide study was to examine the cumulative incidence, survival rates, and the risk of primary endpoints of all-cause mortality, AMI, stroke, HHF, LLA, cancer, and kidney transplantation between undiagnosed CKD (naive) and diagnosed CKD (non-naive) patients (aged > 18 years) following the index date of the first dialysis (2014–2018) among chronic ESKD patients on KRT (2009–2023) in Hungary, using the database of the National Health Insurance Fund (NHIF). We also compared the CKD naive and CKD non-naive groups for exploratory endpoints, including total healthcare costs, major comorbidities, laboratory testing, and medications over 5 years pre-KRT and/or post-KRT.

## 2 Methods

### 2.1 Data source

We retrospectively analyzed data from the NHIF registry, covering adults (over 18 years) who had received dialysis and/or kidney transplantation between 2009 and 2023. Patient-level records were available only from 1 January 2009 onward; therefore, the data extraction for this study covered the period from 1 January 2009 to 30 September 2023 (Supplementary Figure S1). Administrative censoring was applied at 30 September 2023 corresponding to the last available date in the registry at the time of analysis.

Because basic health insurance by the NHIF is mandatory for all residents, it encompasses almost 100% of the Hungarian population. The NHIF database contains information on patient demographics, diagnoses (ICD10 codes; International Classification of Diseases, 10th version), interventions (ICHI codes; International Classification of Health Intervention), prescriptions (ATC codes; Anatomical Therapeutic Chemical), and healthcare services.

Data from all financing registers of outpatient, inpatient, case-financed care (e.g., kidney transplant), dialysis (including P-EPO and dialysis EPO therapy), CT/MR, and dispensed drugs were fully used in this study. All pre-existing data were collected anonymously and presented as aggregated outputs according to the NHIF data protection policy; therefore, informed consent was not required. The study protocol was approved by the Scientific and Research Ethics Committee (TUKEB) (license number: BM/8092–1/2025) and the Ethics Committee of the University of Pécs (approval number: 10120-PTE/2025).

### 2.2 Study design and population

#### 2.2.1 Patient selection

**Figure 1:**
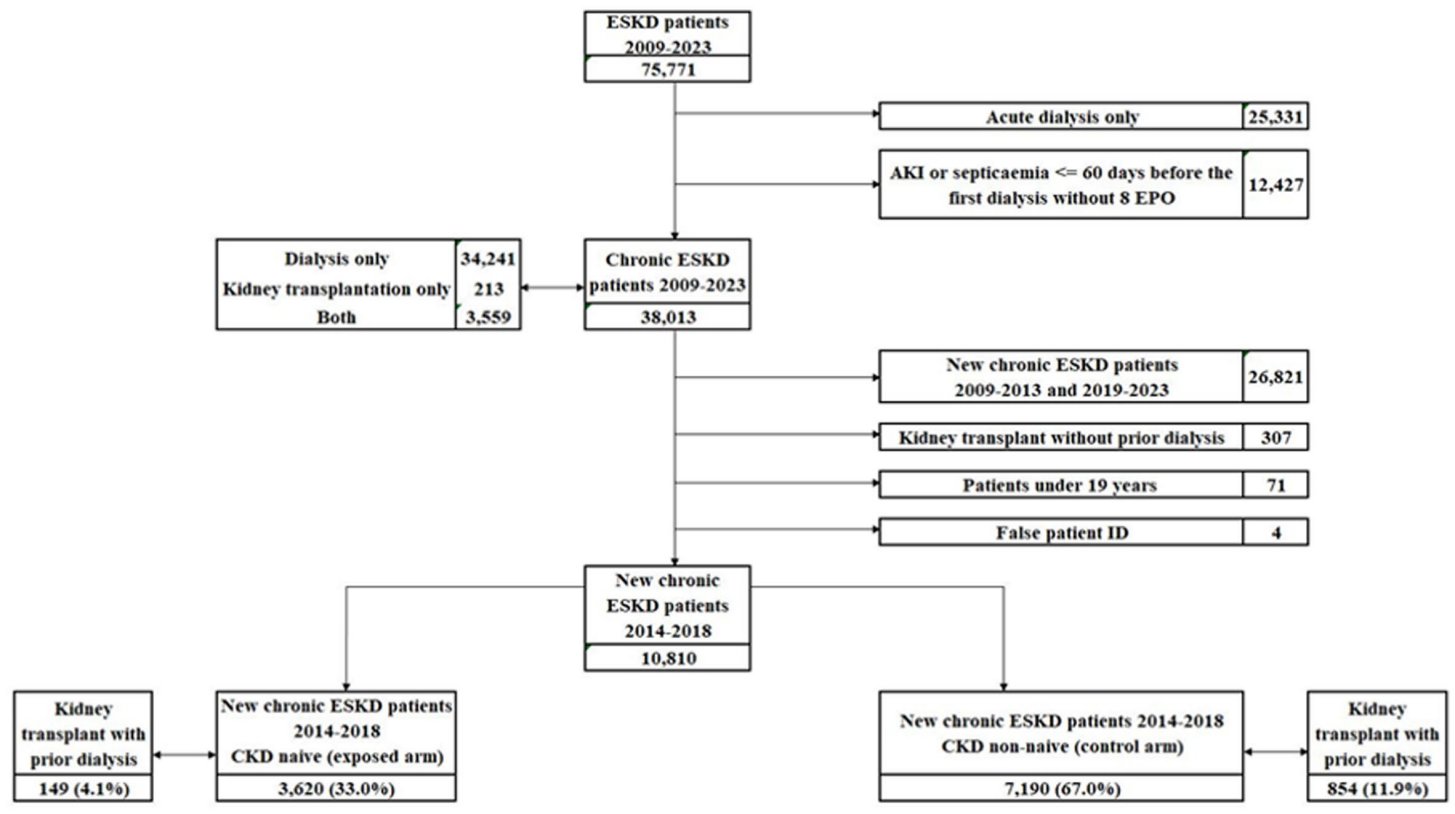
Flow diagram of patient selection. ESKD: end-stage kidney disease; CKD: chronic kidney disease; AKI: acute kidney injury; EPO: erythropoietin.

ESKD patients on KRT were identified for the entire study period (2009–2023) using relevant procedure codes for dialysis and/or kidney transplantation, together with diagnostic codes for CKD and ESKD. Dialysis procedures were defined using ICHI codes for chronic hemodialysis (88531), high-flux dialysis (88585), hemodiafiltration (88604), peritoneal dialysis (88600), and related procedures (88586). Kidney transplantation (living/cadaver donor) was identified using ICHI codes 55551 and 5553. CKD and ESKD diagnoses were defined by ICD10 codes N18-N19. The patient flow and study timeline are shown in Figure 1 and Supplementary Figure S1. The SQL script for all patient selections is presented as Supplementary material 2.

Of this population, we studied ESKD patients who initiated KRT between 1 January 2014 and 31 December 2018 (index period) (Figure 1 and Supplementary Figure S1). We used a 5-year screening period (1 January 2009–31 December 2013) during which no KRT interventions occurred (Supplementary Figure S1). Kidney transplant recipients were excluded if kidney transplantation was their first modality of KRT at the index date or if they had no prior dialysis due to longer life expectancy (4, 20). Kidney transplantation occurring during follow-up was retained and treated as a primary study endpoint.

Patients were eligible for inclusion if they newly started chronic dialysis during the index period (2014–2018) or if they had a kidney transplant with prior dialysis. We categorized them as either undiagnosed CKD naive or diagnosed CKD non-naive at the time of the first dialysis (index date) (Figure 1 and Supplementary Figure S1). Subsequently, the two arms were compared for pre-specified endpoints post-index during 1–6 years of follow-up, and pre-index over 5 years. The follow-up extended from the index date until the first event or death in the main analysis, while patients without events were trailed for the entire follow-up between 1 January 2014 and 31 December 2019 (to exclude the impact of COVID). Sensitivity analyses extended follow-up to 31 December 2023 (Supplementary Figure S1).

#### 2.2.2 Exclusion criteria

Patients who required acute dialysis, or had acute kidney injury (AKI; ICD10 N17), or septicemia (ICD10 A41) within 60 days before the first dialysis were excluded (Figure 1). However, patients who had received eight or more P-EPO prescriptions before the last occurrence of N17 or A41 were retained, as repeated EPO therapy indicates scheduled nephrological visits due to underlying CKD with renal anemia. Additionally, patients with fewer than eight EPO prescriptions before dialysis initiation were excluded (Figure 1). Chronic ESKD patients who had their first dialysis before (2009–2013) or after (2019–2023) the index period were also excluded (Figure 1).

#### 2.2.3 Definition of chronic dialysis

Initial data exploration indicated frequent use of the acute dialysis code (ICHI 88530) in patients with CKD/ESKD. To minimize misclassification due to coding errors, a time-based definition was applied to distinguish chronic dialysis from acute dialysis.

Chronic dialysis was defined if any of the following criteria were met: (1) at least one record of chronic dialysis codes (88531, 88585, 88604, or 88600); (2) the acute dialysis code (88530) was recorded, but the interval between the first and last dialysis sessions exceeded 30 days; or (3) the patient died within 30 days after the first dialysis session (to eliminate immortal bias) (Supplementary material 2).

Cases were considered acute dialysis if only the acute dialysis code (88530) was recorded, treatment lasted less than 30 days, and the patient remained alive after 30 days following the first dialysis. Chronic dialysis in this study was therefore defined as dialysis lasting longer than 30 days (Supplementary material 2).

#### 2.2.4 Erythropoietin therapy

The EPO therapy of pre-dialyzed CKD patients was identified using the ICHI code 59572 (Supplementary material 2). In Hungary, P-EPO use is strictly regulated within the NHIF’s dialysis financial register and is fully reimbursed if a nephrologist specifically prescribes the therapy, typically during monthly follow-up visits for the treatment of renal anemia in patients with advanced CKD. Prescriptions are generally recorded under ICD10 codes N18-N19 (CKD) or D63.80 (anemia in other chronic diseases). Therefore, the presence of P-EPO therapy was considered an indicator of ongoing nephrological care.

Because P-EPO is prescribed at regular monthly visits, it was assumed to represent sustained nephrological management. As prescriptions are recorded as 4-week treatments in the registry, treatment duration was not further examined.

#### 2.2.5 Definition of CKD naive and CKD non-naive patients

Patients were categorized according to evidence of prior CKD diagnosis (based on ICD10 code) and nephrological care (based on P-EPO therapy). Different temporal thresholds (0, 30, 60, and 90 days) for defining CKD status were explored during preliminary analyses. After reviewing raw data and exploratory analyses, a 60-day threshold was selected for the final model.

CKD non-naive (diagnosed) patients were defined as those who had at least two ICD10 codes for CKD (N18-N19) recorded on separate days, with the first code occurring more than 60 days before the index date (the second code could occur within 60 days preceding dialysis). Alternatively, patients were also classified as CKD non-naive if they received at least one prescription of P-EPO more than 60 days before dialysis initiation, indicating regular nephrological care. We applied at least two CKD diagnosis codes to increase diagnostic reliability and reduce the likelihood of miscoding (Supplementary material 2).

Patients were considered CKD naive (undiagnosed) if they had no reported CKD (ICD10 N18-19) and had not received P-EPO therapy more than 60 days before the index date, indicating absence of a prior recognized CKD diagnosis or regular nephrology follow-up.

### 2.3 Propensity score subclassification

Propensity score (PS) subclassification was used to balance confounders between CKD naive and CKD non-naive groups across six equally sized clusters (1,802 patients per subclass) (Supplementary Figure S2). Risk factors included demographics, 21 comorbidities (e.g., diabetes, hypertension, and CVDs), and 32 medications (e.g., antidiabetics, antihypertensives, antiplatelets, and statins) (Supplementary Tables S1–S3). Definitions of all examined confounders are detailed in Supplementary Tables S4, S5. Although the PS method can only balance measured confounders based on the available data of reported comorbidities and medications, matching of the two study arms was optimal across the 56 examined variables (Supplementary Tables S1–S3).

### 2.4 Study endpoints

Primary endpoints included death from any cause, AMI with PCI (percutaneous coronary intervention), stroke (by CT/MR), HHF, LLA, cancer, and kidney transplantation (definitions are listed in Supplementary Table S6). MACE (major adverse cardiovascular events) included all-cause mortality, because cause-specific deaths were unavailable from NHIF. A primary composite endpoint consisting of MACE, HHF, LLA, cancer, and kidney transplantation was also evaluated. Cumulative incidence, survival rates, and risk comparisons of primary endpoints were assessed post-index (for a maximum of 72 months). Patients with pre-index events were not analyzed. Any events occurring after the first event were excluded. Patients without events were followed for the entire follow-up period.

Secondary endpoints were the prevalence of comorbidities (based on a single primary or secondary ICD-diagnosis from outpatient or inpatient visits), laboratory testing rates (based on ICHI codes for ambulatory albuminuria and serum creatinine measurements), and drug therapy (based on ATC codes) in the 5 years preceding the index date.

Tertiary endpoints involved total healthcare costs (in Euro per capita) over 5 years pre-index and post-index, categorized by financial registers (i.e., inpatient care, outpatient care, drug therapy, dialysis, or CT/MR). Costs were analyzed for a full (non-calendar fiscal) year.

### 2.5 Statistical analyses

We calculated the standardized mean difference (SMD) for baseline parameters before and after PS subclassification. Descriptive statistics are presented as frequencies and corresponding percentages for pre-index comorbidities and drug usage. These descriptives were unadjusted.

Time-to-event analyses were conducted to evaluate primary study endpoints. We calculated the number of events and censorings (administrative at 30 September 2023), and cumulative incidence. We distinguished between two analytical objectives: (1) descriptive estimation of cumulative incidence reflecting real-world (observable) risk using the Aalen-Johansen method, and (2) comparison of the risk of endpoints between the CKD naive and CKD non-naive groups, reflecting underlying (biological) effects using a cause-specific Cox regression model.

Death is an absorbing event. To estimate real-world risk, we used the Aalen–Johansen method for non-fatal events, with death as the competing event. Death was considered a competing event because it precludes the occurrence of non-fatal events (HHF, AMI, stroke, LLA, and cancer) and is likely associated with their risk due to shared underlying factors. The Kaplan–Meier method treating death as censoring may yield biased estimates of cumulative incidence for non-fatal events due to informative censoring. We therefore used the Aalen–Johansen estimator to estimate the cumulative incidence of non-fatal endpoints, which appropriately accounts for competing risks. Kaplan–Meier curves were applied for all-cause mortality and for the composite endpoint that included death. In the competing risk analyses, the first occurrence of each endpoint (AMI, stroke, HHF, LLA, cancer, and kidney transplantation) was evaluated within the respective study arm.

For comparative analyses between study arms, we estimated hazard ratios (HRs) using Cox proportional hazards models. These models target the association between CKD naive or CKD non-naive status and event, reflecting the underlying (biological) effect rather than the real-world cumulative incidence. We estimated cause-specific HRs for each primary endpoint, treating competing events accordingly. Models were adjusted for baseline hazard differences across PS subclasses and age groups. The hazard ratio of the composite primary endpoint decreased in a time-dependent manner after dialysis, becoming constant at 18 months post-KRT. As the proportional hazard assumption was not satisfied, we applied a linear time-dependent covariate from 1 month to 18 months. We also calculated the cause-specific HRs, assuming the same time-dependence for each possible endpoint. We used parametric bootstrap with 1 M repetitions to calculate the confidence interval (CI) and the *p*-value for the first 18 months.

Changes in the age distribution of the two arms between post-index 1 month and 18 months were analyzed by binomial regression. The proportion of patients with a specific comorbidity and drug type before dialysis was compared by chi-squared test. Total cost was compared by Welch’s *t*-test for each year.

We used R version 4·2·2 with the following packages: survival 3·7–0, tidycmprsk 1·1·0, and MatchIt 4·7·0.

## 3 Results

### 3.1 Study population

The total number of all ESKD patients who received any KRT was 75,771 in 2009–2023 (period prevalence) (Figure 1). We excluded 25,331 patients (33.4%) who required acute dialysis and 12,427 patients (16.4%) who did not fulfill the criteria for chronic ESKD due to AKI, septicemia, or missing ≥8 P-EPO. The remaining chronic ESKD patients on KRT totaled 38,013 (50.2%), of whom 34,241 (90.0%) had dialysis, 213 (0.6%) received a kidney transplant (irrespective of prior dialysis), and 3,559 (9.4%) had both modalities (Figure 1). We subsequently excluded those who started dialysis before (2009–2013) or after (2019–2023) the index period (*n* = 26,821), kidney transplant patients without prior dialysis (*n* = 307), and those who were not adults (*n* = 71) or had false IDs (*n* = 4) (Figure 1).

The study population included 10,810 new chronic ESKD patients who initiated dialysis in 2014–2018 (Figure 1). Among them, 3,620 were CKD naive (33.0%), and 7,190 were CKD non-naive (67.0%) patients. More kidney transplant patients (with prior dialysis) were observed in the CKD non-naive arm (*n* = 854; 11.9%) than in the CKD naive arm (*n* = 149; 4.1%), with dialysis being the dominant modality in both arms (Figure 1).

### 3.2 CKD naive and CKD non-naive patients before and after PS subclassification

PS subclassification improved the SMD values of sex and age groups within the 10% cutoff (Supplementary Table S1 and Supplementary Figure S3). Male dominance was observed (CKD naive: 58.8%; CKD non-naive: 55.0%). Most patients were aged 60–79 years (CKD naive: 56.3%; CKD non-naive: 55.1%) (Supplementary Table S1).

Dissimilar SMD values of comorbidities after PS subclassification also fell below 10% (Supplementary Table S2 and Supplementary Figure S4). The prevalence of prior comorbidities (in 5 years pre-KRT) was higher in the CKD non-naive versus the CKD naive arm (Supplementary Figures S5, S6), including hypertension (92.9% vs. 59.6%, *p* < 0.001), type 1 diabetes (2.0% vs. 0.6%, *p* < 0.001), type 2 diabetes (T2DM) (46.7% vs. 26.2%, *p* < 0.001), polycystic kidney disease (PKD) (5.9% vs. 0.7%, p < 0.001), glomerulonephritis (11.2% vs. 2.1%, *p* < 0.001), AMI (4.9% vs. 3.7%, *p* = 0.005), stroke (7.1% vs. 6.0%, *p* = 0.03), HHF (38.2% vs. 23.7%, *p* < 0.001), and LLA (4.5% vs. 3.1%, *p* = 0.001), except for cancer (18.2% vs. 26.3%, *p* < 0.001) (Supplementary Figures S5, S6).

### 3.3 Primary endpoints

#### 3.3.1 Unadjusted data

The number of analyzed patients (without prior events) was 2,218 (61%) in the CKD naive arm [administratively censored *n* = 1,584 (34%)], and 4,615 (64%) in the CKD non-naive arm [administratively censored *n* = 403 (18%)]. The total number of events amounted to 1,815 in the CKD naive arm and 3,031 in the CKD non-naive arm.

**Figure 2:**
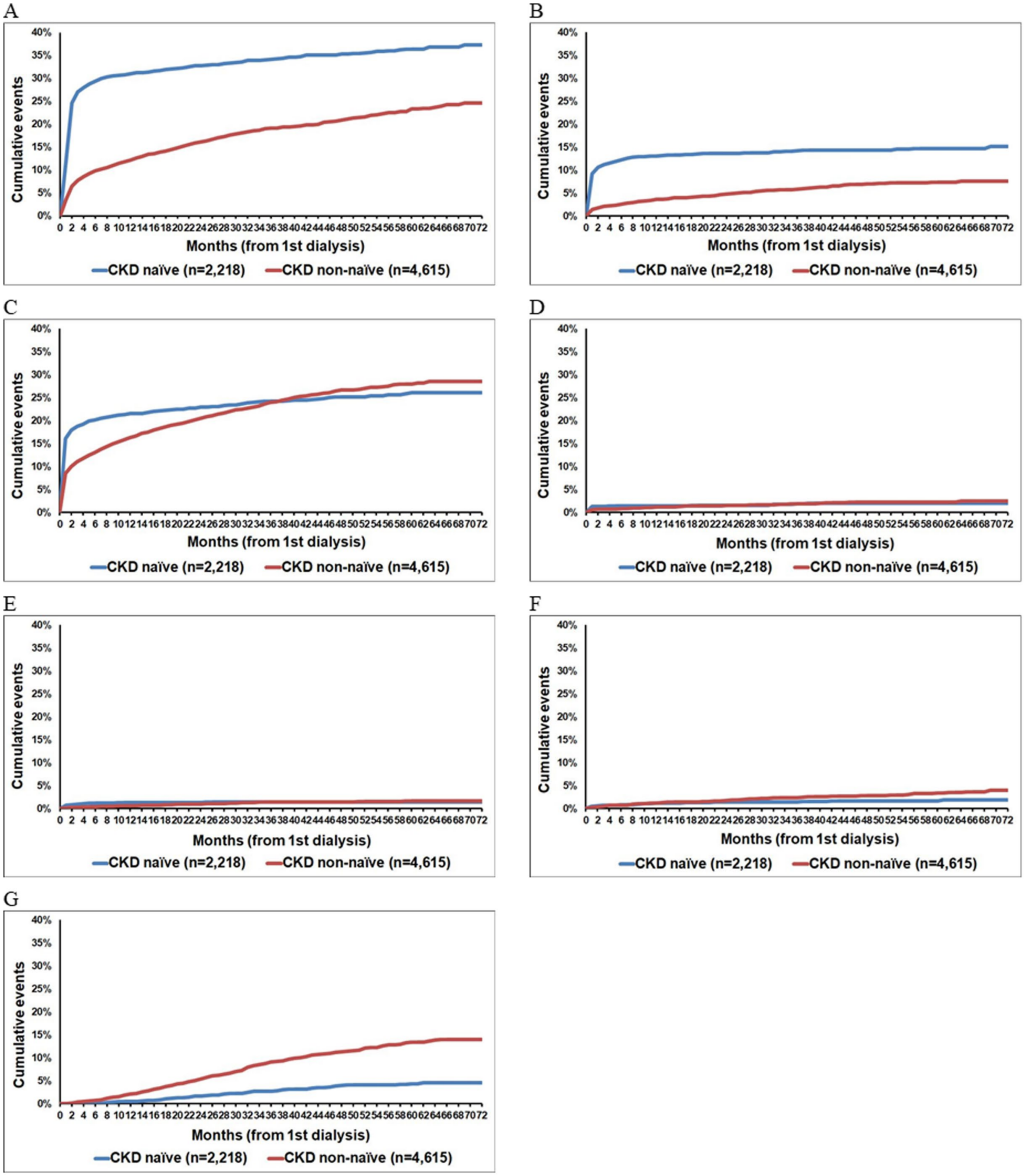
Cumulative incidence of the primary endpoints from the time-to-first event analyses in the CKD naive arm (*n* = 2,218) and the CKD non-naive arm (*n* = 4,615) during the 72-month follow-up after the first dialysis. Primary endpoints were **(A)** all-cause mortality, **(B)** cancer, **(C)** hospitalization for heart failure, **(D)** acute myocardial infarction with percutaneous coronary intervention, **(E)** stroke (with CT/MR), **(F)** lower limb amputation, and **(G)** kidney transplantation. Data are unadjusted for the different propensity score subclassification hazards (no differences in hazards and statistics provided).

The cumulative incidence was higher at 72 months in CKD naive patients for all-cause mortality (37.3% vs. 24.6%) and new cancers (15.2% vs. 7.6%). Both remained higher during the 72 month follow-up compared to CKD non-naive patients (Figures 2A,B). Post-KRT event rates increased sharply: 27% of CKD naive patients died (vs 7.8% of CKD non-naive patients) and 11.2% of CKD naive patients had cancer (vs 2.1% of CKD non-naive patients) at 3 months post-KRT (Figures 2A,B).

Hospitalization for heart failure also increased abruptly, particularly among CKD naive patients (18.8% vs. 11.1% at 3 months). HHF then displayed higher rates in the CKD non-naive arm, reaching that of the CKD naive arm at 36 months post-KRT (Figure 2C). HHF was then reversed at 72 months, found modestly more frequent in the CKD non-naive arm (28.5% vs. 26.1% of CKD naive arm) (Figure 2C).

The cumulative incidence of AMI (CKD naive: 2% vs. non-naive: 2.5%), stroke (CKD naive: 1.5% vs. non-naive: 1.7%), and LLA (CKD naive: 1.9% vs. non-naive: 4%) was relatively low and remained comparable in the two arms at 72 months of follow-up (Figures 2D–F).

Kidney transplantation showed upward trends in both arms, but the rate was higher in CKD non-naive versus CKD naive patients (14% vs. 4.6%) at 72 months of follow-up (Figure 2G).

**Figure 3:**
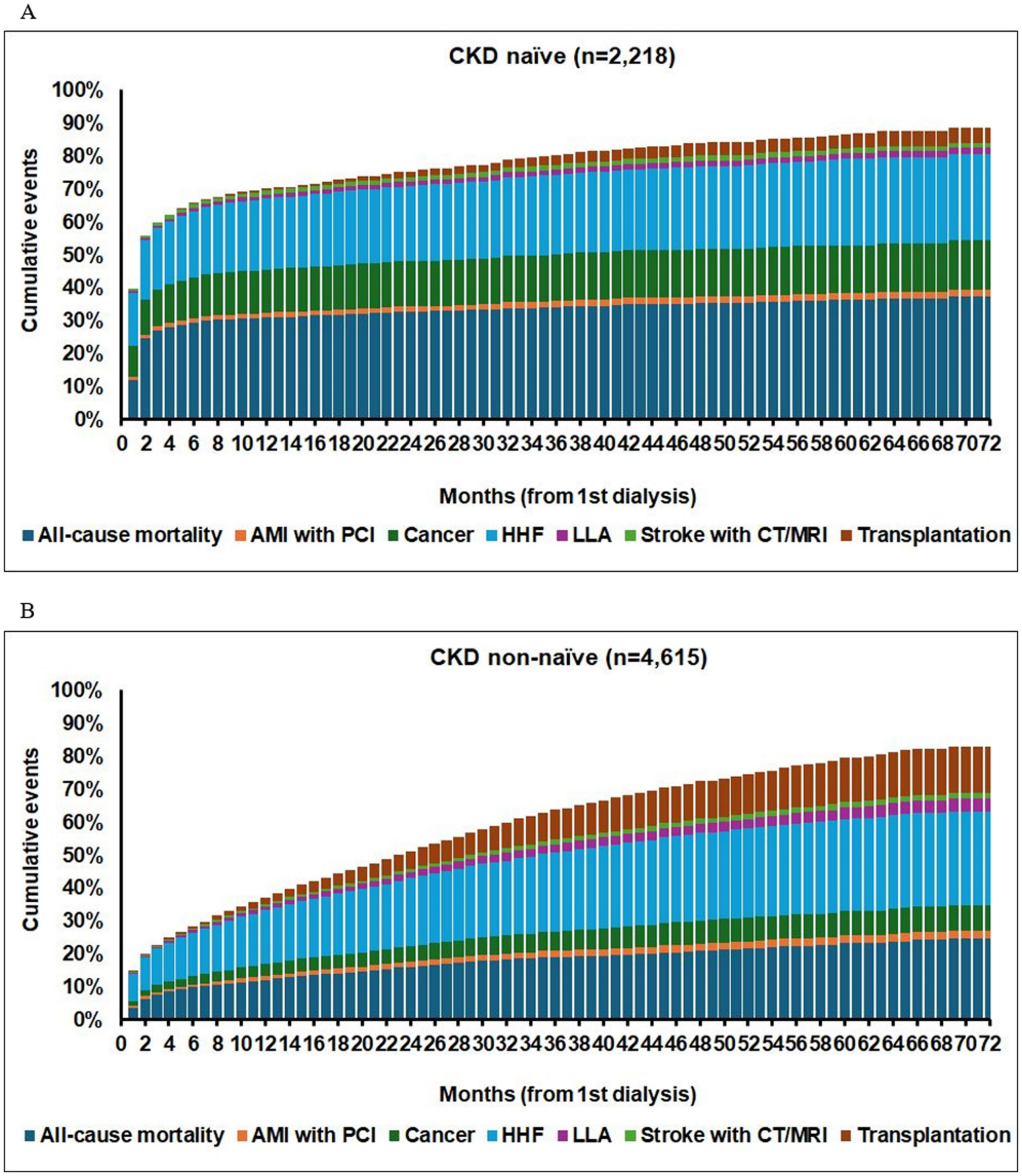
Integrated summary of the primary endpoints in the **(A)** CKD naive arm (*n* = 2,218) and **(B)** CKD non-naive arm (*n* = 4,615) during the 72-month follow-up after the first dialysis. Results are outputs of the cumulative event rates from the time-to-first event analyses. Total number of events were *n* = 3,031 in the CKD non-naive arm, and *n* = 1,815 in the CKD naive arm. The top 3 events were all-cause death, HHF (hospitalization for heart failure), and cancers in the CKD naive arm. The top 3 events were HHF, all-cause death, and kidney transplantation in the CKD non-naive arm. Data are unadjusted for the different propensity score subclassification hazards (no differences in hazards and statistics provided). AMI with PCI: acute myocardial infarction with percutaneous coronary intervention; LLA: lower limb amputation.

All-cause death was the most frequent event for CKD naive patients, followed by HHF and cancers, all of which remained highly frequent during the entire 72 month follow-up, but specifically shortly after post-KRT (Figure 3A and Supplementary Figure S7A). In CKD non-naive patients, HHF was the leading event, followed by all-cause death and kidney transplantation (Figure 3B and Supplementary Figure S7B).

**Figure 4:**
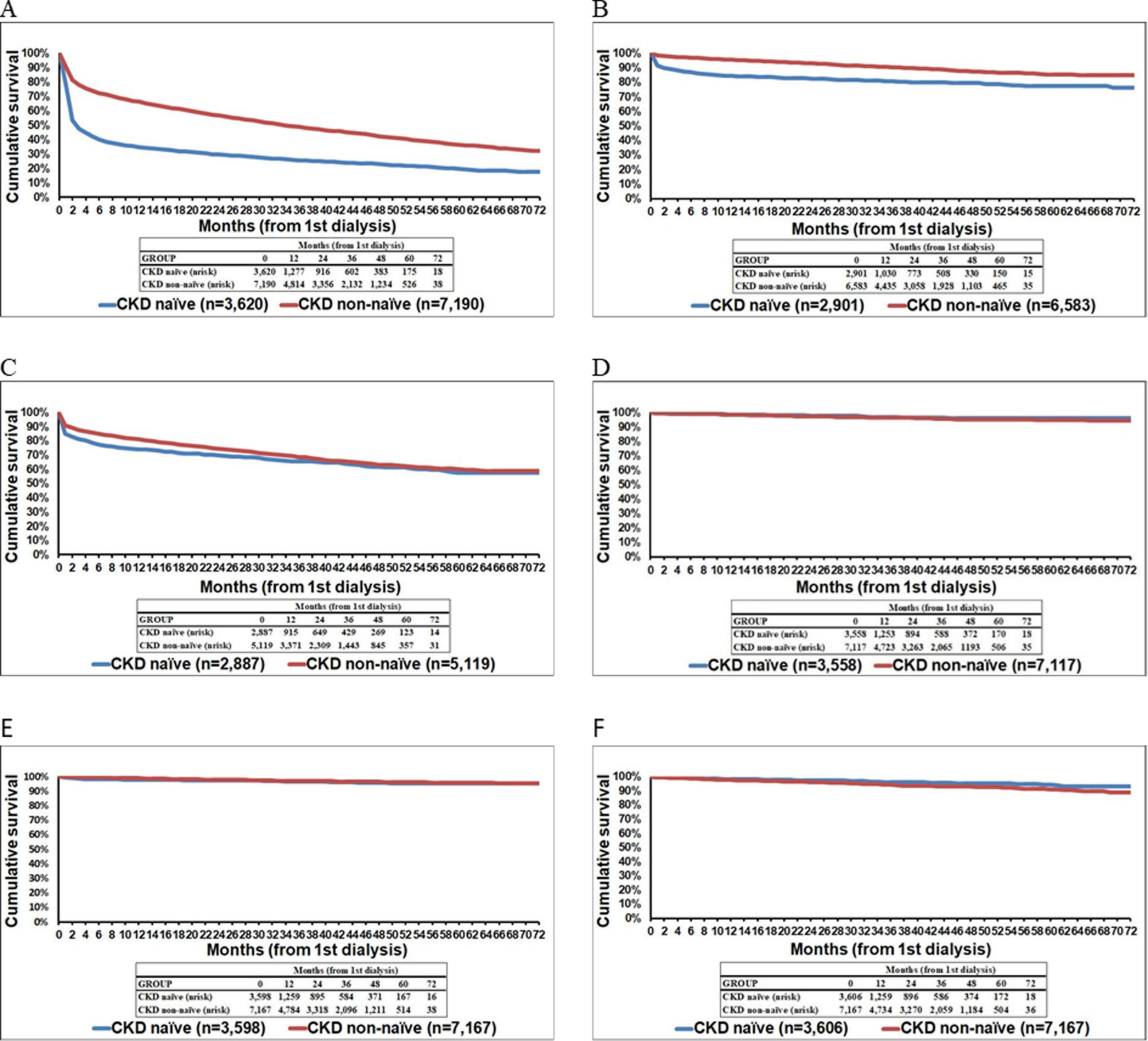
Kaplan–Meier survival curves for **(A)** all-cause mortality and disease-free survivals for **(B)** cancer, **(C)** hospitalization for heart failure, **(D)** acute myocardial infarction with percutaneous coronary intervention, **(E)** stroke (with MR/CT), and **(F)** lower limb amputation during the 72-month follow-up after the first dialysis in the CKD naive arm versus the CKD non-naive arm. Numbers of patients at risk (nrisk) are shown for both arms. Data are unadjusted for the different propensity score subclassification hazards (no differences in hazards and statistics provided).

The overall survival of CKD naive patients declined more rapidly post-KRT (47.5% vs. 78.1% for CKD non-naive patients at 3 months) and remained lower during the 72 month follow-up (Figure 4A). Median survival was markedly shorter for CKD naive (3 months) than for CKD non-naive patients (34 months). In the sensitivity analysis, the survival curve of the CKD non-naive arm diverged and decreased to a greater extent, approximating that of the CKD naive arm during the 116 month follow-up (Supplementary Figure S8).

Cancer-free survival was also lower in the CKD naive versus the CKD non-naive arm throughout the 72 month follow-up, ranging between 88.9–76.1% versus 97.4–85.0% after 3 months post-KRT (Figure 4B).

HHF-free survival of CKD naive patients decreased more initially post-KRT (81.3% vs. 87.7% for CKD non-naive patients at 3 months) and was lower during the follow-up (57.4% vs. 58.6% at 72 months) (Figure 4C).

Higher survival rates were found at 3 months and 72 months for AMI (CKD naive: 98.9–95.9% vs. non-naive: 99.1–94.1%) and stroke (CKD naive: 98.6–95.4% vs. non-naive: 99.5–95.1%) in both arms post-KRT (Figures 4D,E). LLA-free survival was lower in the CKD non-naive than the CKD naive arm after 36 months (89.1% vs. 93.3% at 72 months) (Figure 4F).

#### 3.3.2 Adjusted, PS subclassification-matched data

**Figure 5:**
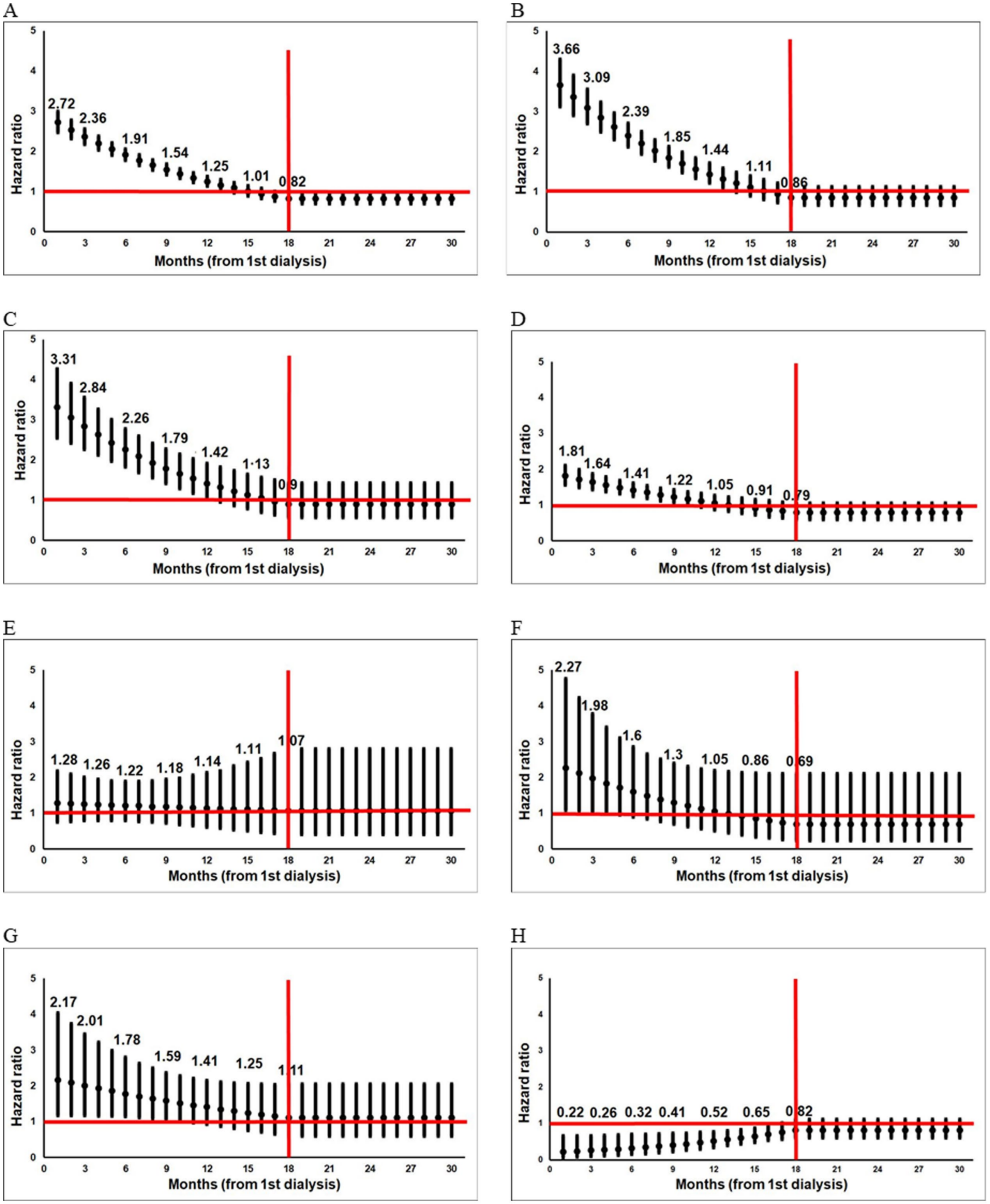
Results of Cox regression analyses to compare the risk of the **(A)** primary composite endpoint, **(B)** all-cause mortality, **(C)** cancer, **(D)** hospitalization for heart failure (HHF), **(E)** acute myocardial infarction with percutaneous coronary intervention (AMI with PCI), **(F)** stroke, **(G)** lower limb amputation (LLA), **(H)** kidney transplantation in the CKD naive arm versus the CKD non-naive arm during the 30-month follow-up after the first dialysis. The primary composite endpoint consisted of MACE (major cardiovascular events, including all-cause mortality, AMI with PCI, stroke), cancer, HHF, LLA, and kidney transplantation. Data were corrected for the different hazards of the propensity score subclassification and age groups. Time-dependent changes in the hazard ratios (HR) became constant at 18 months post-index.

Cox regression showed that the risk of the primary composite endpoint (MACE, HHF, cancer, LLA, and kidney transplantation) was almost 3-fold higher in the CKD naive arm [HR: 2.72 (CI: 2.47–2.99), *p* < 0.001] at 1 month post-KRT, which remained significantly higher at 6 months [HR: 1.91 (CI: 1.77–2.06), *p* < 0.001] and 12 months [HR: 1.25 (CI: 1.13–1.38), p < 0.001] compared to the CKD non-naive arm (Figure 5A). We found a reversed risk at 18 months, being 18% lower for CKD naive patients [HR: 0.82 (CI: 0.70–0.95), *p* = 0.01], suggesting that CKD naive patients were possibly dropped out due to higher early mortality (Figure 5A).

All-cause mortality risk of CKD naive patients was almost 4-fold higher [HR: 3.66 (CI: 3.12–4.29), *p* < 0.001] at 1 month post-KRT, which was significantly higher at 6 months [HR: 2.39 (CI: 2.11–2.71), *p* < 0.001] and 12 months [HR: 1.44 (CI: 1.21–1.71), p < 0.001] than that of CKD non-naive patients (Figure 5B). Death risk at 18 months did not differ between the groups [HR: 0.86 (CI: 0.66–1.13), *p* = 0.29] (Figure 5B).

We found a 3-fold higher cancer risk for CKD naive patients [HR: 3.31 (CI: 2.55–4.29), *p* < 0.001] at 1 month post-KRT, which persisted at 6 months [HR: 2.26 (CI: 1.83–2.78), *p* < 0.001] and 12 months [HR: 1.42 (CI: 1.06–1.92), *p* < 0.001] compared with CKD non-naive patients (Figure 5C). Cancer risk was similar at 18 months between the arms [HR: 0.90 (CI: 0.57–1.43), *p* = 0.66] (Figure 5C).

HHF risk was significantly higher for CKD naive versus CKD non-naive patients at 1 month [HR: 1.81 (CI: 1.55–2.11), *p* < 0.001] and 6 months [HR: 1.41 (CI: 1.24–1.61), *p* < 0.001] post-KRT, with no difference at 12 months [HR: 1.05 (CI: 0.87–1.27), *p* = 0.58] and afterward [18 months: HR: 0.79 (CI: 0.59–1.05), *p* = 0.1] (Figure 5D).

AMI risk did not differ between the arms post-KRT [1 month: HR: 1.28 (CI: 0.75–2.18), *p* = 0.36; 6 months: HR: 1.22 (CI: 0.79–1.89), *p* = 0.38; 12 months: HR: 1.14 (CI: 0.61–2.14), *p* = 0.67; 18 months: HR: 1.07 (CI: 0.41–2.80), *p* = 0.88] (Figure 5E).

Stroke risk was 2-fold higher for CKD naive versus CKD non-naive patients [HR: 2.27 (CI: 1.08–4.76), *p* = 0.03] at 1 month, which vanished after 4 months post-KRT [6 months: HR: 1.60 (CI: 0.90–2.86), *p* = 0.11; 12 months: HR: 1.05 (CI: 0.51–2.19), *p* = 0.89; 18 months: HR: 0.69 (CI: 0.23–2.11), *p* = 0.52] (Figure 5F).

A higher LLA risk was observed for CKD naive versus CKD non-naive patients at 1 month [HR: 2.17 (CI: 1.17–4.04), *p* = 0.02] and 6 months [HR: 1.78 (CI: 1.14–2.80), *p* = 0.01] post-KRT, with no difference between the arms after 12 months [HR: 1.41 (CI: 0.92–2.15), *p* = 0.11; 18 months: HR: 1.11 (CI: 0.60–2.05), *p* = 0.73] (Figure 5G).

Kidney transplantation probability was consistently lower for CKD naïve patients versus CKD non-naïve patients post-KRT [1 month: HR: 0.22 (CI: 0.07–0.66), *p* = 0.007; 6 months: HR: 0.32 (CI: 0.15–0.70), *p* = 0.004; 12 months: HR: 0.52 (CI: 0.34–0.78), *p* = 0.002; 18 months: HR: 0.82 (CI: 0.60–1.12), *p* = 0.22] (Figure 5H).

**Figure 6:**
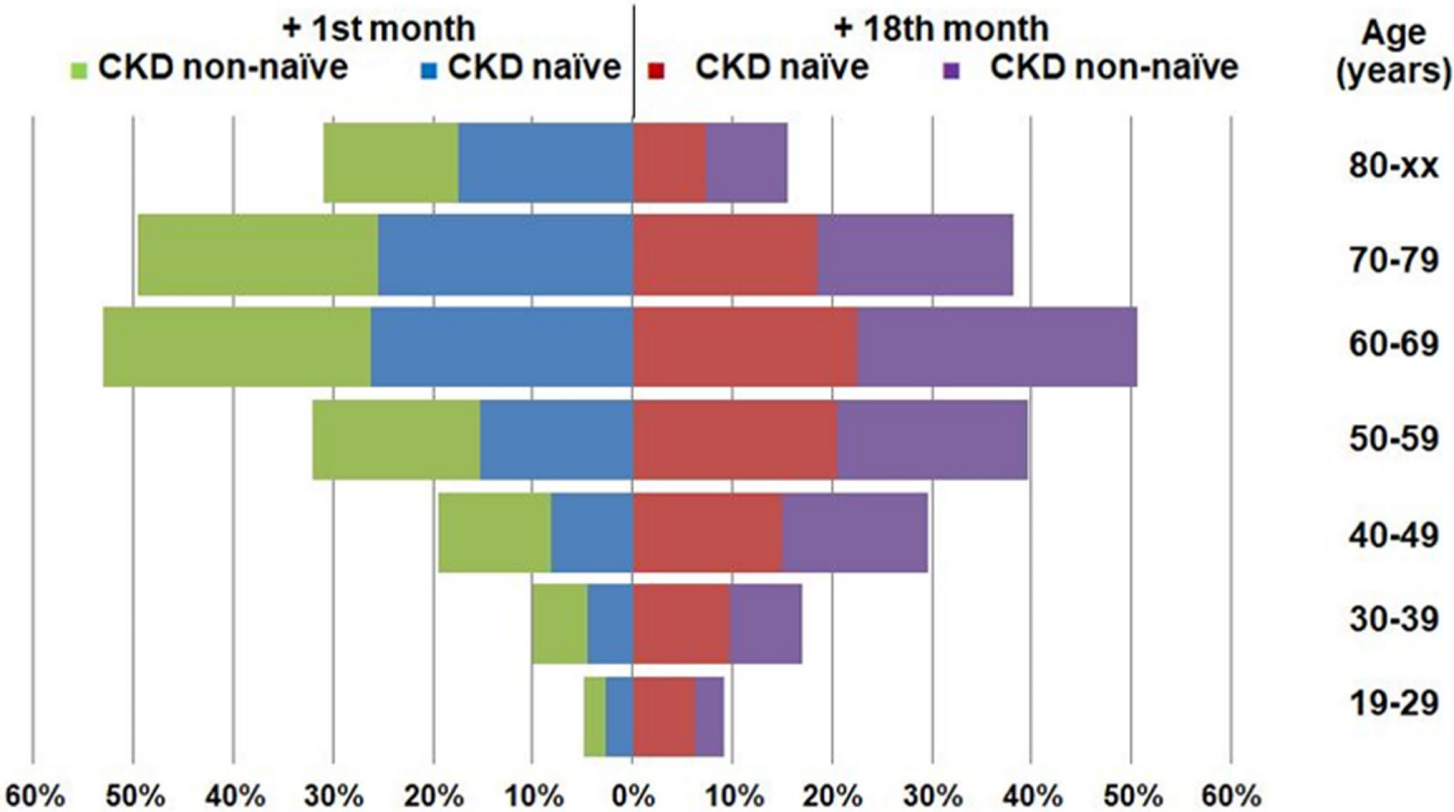
Age distribution of CKD naïve and CKD non-naive patients at 1 month (left) and 18 months (right) after the index date of the first dialysis. Changes in the age distribution of the study arms were significant at 18 months (*p* = 0.0068 versus 1 month) by binomial logistic regression.

The age distribution of the two arms shifted toward younger populations at 1 month versus 18 months post-KRT (*p* = 0.007) (Figure 6). There were decreasing proportions of patients aged over 60 years and increasing proportions of those aged 19–59 years, with a greater extent in the CKD naive arm (Figure 6). CKD non-naïve and CKD naïve patients at 1 month by age groups were 19–29: 2.1% (*n* = 98) and 2.7% (*n* = 60); 30–39: 5.4% (*n* = 248) and 4.6% (*n* = 102); 40–49: 11.3% (*n* = 521) and 8.3% (*n* = 183); 50–59: 16.8% (*n* = 774) and 15.2% (*n* = 338); 60–69: 26.9% (*n* = 1,243) and 26.1% (*n* = 580); 70–79: 24.1% (*n* = 1,110) and 25.6% (*n* = 567); ≥80: 13.5% (*n* = 621) and 17.5% (*n* = 388) (Figure 6). At 18 months, these were 19–29: 3.0% (*n* = 71) and 6.3% (*n* = 36); 30–39: 7.3% (*n* = 176) and 9.7% (*n* = 56); 40–49: 14.5% (*n* = 348) and 15.1% (*n* = 87); 50–59: 19.2% (*n* = 459) and 20.5% (*n* = 118); 60–69: 28.1% (*n* = 673) and 22.6% (*n* = 130); 70–79: 19.7% (*n* = 471) and 18.6% (*n* = 107); ≥80: 8.2% (*n* = 197) and 7.3% (*n* = 42) (Figure 6).

Increased death rates and the secession of older patients during the first 18 months following KRT initiation, mainly from the CKD naive arm, may explain the reduced (or reversed) risk for primary endpoints observed at 18 months post-KRT. Excess mortality was likely due to the increased risk of cancer, HHF, and stroke (Figures 5C,D,F).

### 3.4 Secondary (unadjusted) endpoints

Hypertension, T2DM, anemia, CAD, and HF were among the leading comorbidities in the 5 years pre-KRT in both arms (Supplementary Table S7).

Overall prevalence of hypertension (97 and 75%), T2DM (53 and 29%), anemia (58 and 32%), CAD (54 and 34%), LEAD (44 and 26%), and HF (42 and 27%) were all higher in the CKD non-naive arm compared to the CKD naive arm (Supplementary Figures S9A–G). Crude prevalence of all comorbidities was consistently higher for CKD non-naive patients (*p* < 0.001) in each of the five index years before dialysis (Supplementary Figures S9A–G). Notably, all of these comorbidities were increasingly diagnosed closer to dialysis in both groups, with a peak in the last year before KRT initiation (Supplementary Figures S9A–G).

CKD diagnosis was present in 38% of CKD naive patients at 1 year (i.e., within a maximum of 60 days) before KRT initiation based on our predefined criteria (Supplementary Figures S9D). Of the CKD non-naive patients, 36% had a diagnosis at 5 years and 95% at 1 year pre-KRT (without P-EPO) (Supplementary Figure S9D).

Kidney function and albuminuria testing rates were higher for CKD non-naive patients in each year pre-KRT (*p* < 0.001) (Supplementary Figure S10). Kidney function tests were increasingly performed in both groups from 5 years to 1 year pre-KRT (from 47 to 79% for CKD naive, and 77 to 97% for CKD non-naive patients). Albuminuria tests occurred infrequently in all pre-KRT years (overall testing was 14% for CKD naive, and 54% for CKD non-naive patients) (Supplementary Figure S10).

Furosemide was the most dispensed drug in both groups; potassium chloride and amoxicillin–clavulanic acid were also commonly administered (Supplementary Tables S8, S9). P-EPO was only applied in 3% of CKD naive patients at 1 year (i.e., during 60 days) before KRT initiation (vs 47% of CKD non-naive patients) (Supplementary Table S9 and Supplementary Figure S11A). Furosemide was used more frequently amongst CKD non-naive patients in each year pre-KRT (*p* < 0.001) (Supplementary Figure S11B). More CKD non-naive patients were given angiotensin-converting enzyme inhibitors (22.9–31.4% vs. 14.5–23.1%), spironolactone (24.8% vs. 17.6%), and statins (30.7–46.3% vs. 21.5–25.9%) than CKD naive patients (Supplementary Tables S8, S9).

### 3.5 Tertiary (unadjusted) endpoints

**Figure 7:**
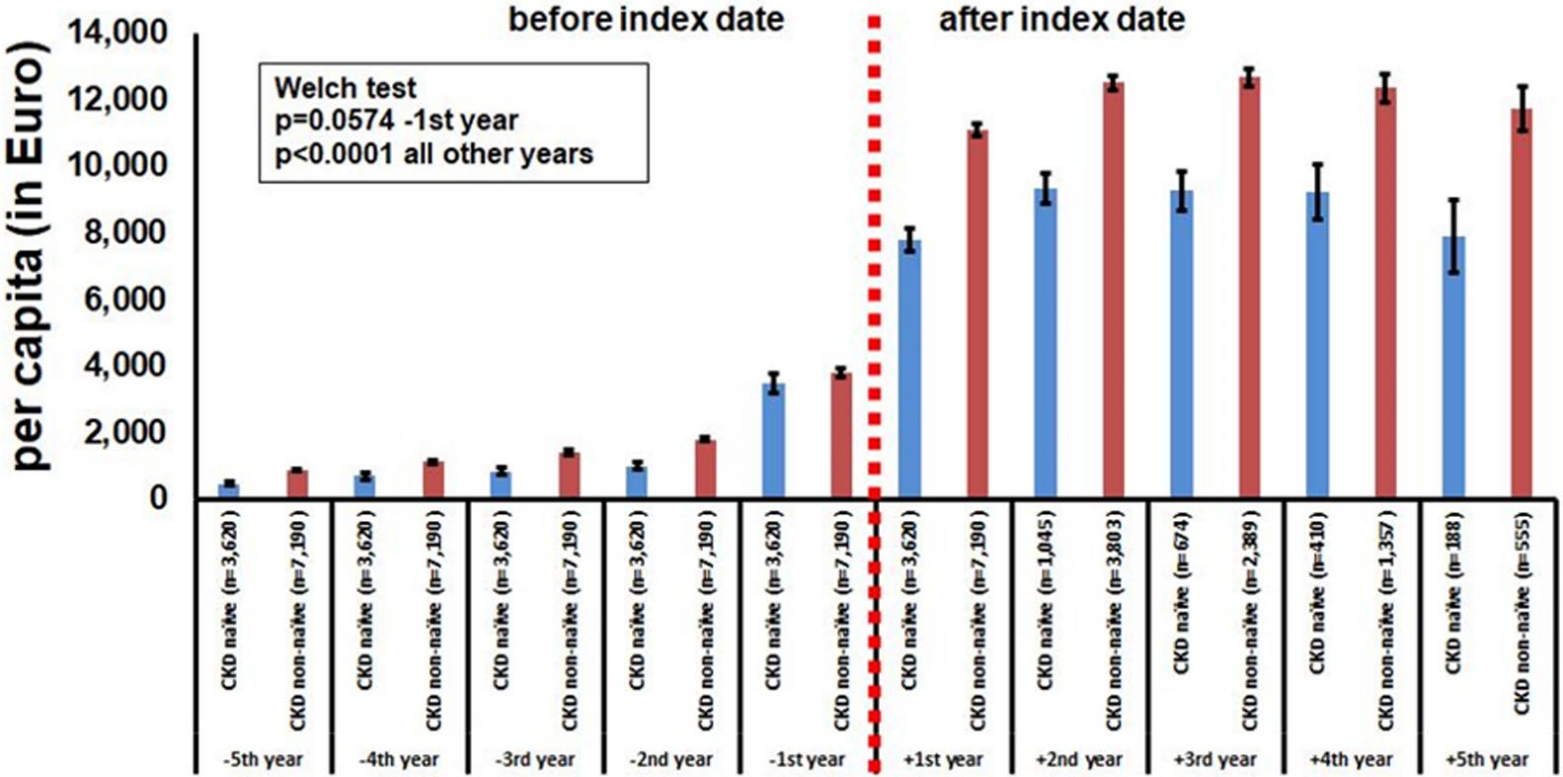
Average total healthcare costs (per capita in Euro) comparing CKD naïve and CKD non-naive arms over 5 years before and after the index date of the first dialysis. CKD naïve versus CKD non-naive: *p* < 0.0001 and *p* = 0.0574 (Welch’s *t*-test); Data are given with 95% confidence interval (CI). The number of patients at risk decreases progressively from the second year after the index date.

**Figure 8:**
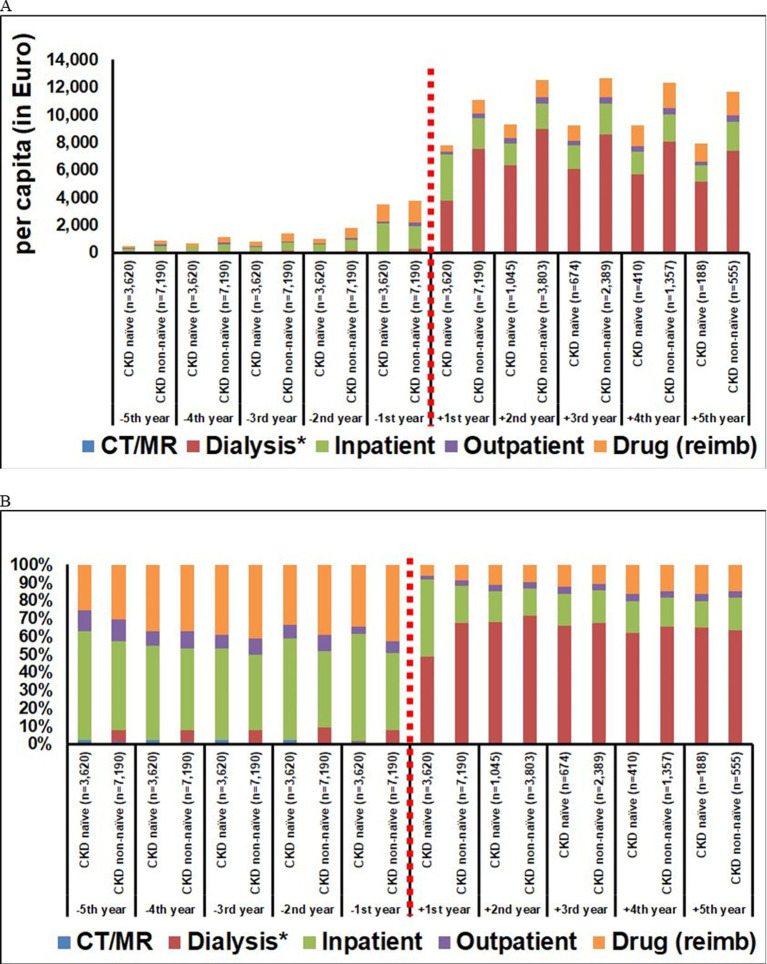
Based on financial registers, **(A)** absolute and **(B)** percentage distribution of the average total healthcare costs (per capita in Euro) comparing CKD naïve and CKD non-naive arms over 5 years before and after the index date of the first dialysis. *Dialysis register includes pre-dialysis erythropoietin (P-EPO) therapy, dialysis erythropoietin (EPO) therapy, and dialysis care.

Total (*per capita*) healthcare costs for CKD non-naive patients were significantly higher each year before and after dialysis (*p* < 0.001), except 1 year pre-KRT (*p* = 0.06) (Figure 7). Before dialysis, inpatient care spending contributed most to expenses, followed by drug therapy in all pre-index years, while dialysis care was the major expenditure post-KRT in both arms (Figures 8A,B).

Expenditures increased gradually toward KRT in both arms, with the greatest increase at 1 year pre-KRT (Figure 7). Costs increased more intensely (7.5-fold) in the CKD naive arm (from 460€ to 3,486€) compared to a 4.5-fold increase in the CKD non-naive arm (from 852€ to 3,792€) between 5 years to 1 year pre-KRT (Figure 7). Expenses escalated post-KRT and persisted high for both CKD naive (7,787-9,322€) and CKD non-naive (11,094-12,663€) patients (Figure 7).

Drug therapy costs were consistently higher for CKD non-naive versus CKD naive patients in all 5 years pre-KRT and post-KRT, including 1 year before dialysis [1,622€ (43%) vs. 1,209€ (35%)] (Figures 8A,B). Relative costs tended to be equalized at 2 years post-KRT, but were marginally higher for CKD naive (11–16%) than CKD non-naive (10–15%) patients (Figure 8B).

Hospitalization costs increased markedly for CKD naive patients pre-KRT, exceeding that of the CKD non-naive patients at 1 year before dialysis [2,094€ (60%) vs. 1,634€ (43%)] (Figures 8A,B). Hospitalization costs of CKD naive versus CKD non-naive patients remained higher at 1 year post-KRT [3,350€ (43%) vs. 2,249€ (20%)], especially relative to the cost of dialysis [3,774€ (48%) and 7,496€ (68%)] (Figures 8A,B). These differences disappeared at 5 years post-KRT (Figure 8B).

## 4 Discussion

In this nationwide study, we showed that undiagnosed CKD (naive) patients compared to diagnosed CKD (non-naive) patients have a higher risk for all-cause mortality (3.7 times), cancers (3.3 times), HHF (1.8 times), stroke (2.3 times), and LLA (2.17 times) at 1 month after KRT initiation. Undiagnosed CKD patients had a lower likelihood for kidney transplantation by 78–48% during the first post-dialysis year, and showed lower kidney transplantation rates (3% versus 10%) compared to diagnosed CKD patients over the observed 6 years post-dialysis.

The risks faded over time post-KRT and were no longer different between the two cohorts after 18 months. In fact, we found an 18% lower risk for the primary composite endpoint (of MACE, HHF, cancer, LLA, and kidney transplant) in undiagnosed CKD patients. This reversed risk—driven by cancer, HHF, and stroke—was attributed to higher early mortality in a more vulnerable elderly population (21), resulting in a younger survival cohort, particularly among undiagnosed CKD naive patients. Median survival of undiagnosed CKD patients (3 months) was markedly (11-fold) shorter compared to diagnosed patients (34 months).

A primary care-based retrospective study including 10,551 patients with stage 3–5 CKD showed 29% higher mortality for unregistered CKD patients, but it failed to establish an increased risk for incident CVDs (HR: 0.92) (22).

A recent nationwide study showed that 74% of new KRT patients were diagnosed late for CKD (within 6 months pre-KRT) and had an 18% higher mortality risk and death rates (34% versus 23%) compared to those with an early diagnosis during the median follow-up of 537 days (20). The survival benefit for early diagnosed patients was 6% at 6 months post-KRT (20). Crude mortality rates were similar, but the risk difference was quite moderate compared with our results, although we used different criteria to define undiagnosed/diagnosed CKD. They also found increased kidney transplantation rates for early diagnosed patients (14% versus 11.4%), associated with an 81% risk reduction for death. Additional data for cancer or CVDs have not been reported (20).

Late referral of CKD patients is linked to increased mortality (23, 24). Predictors of unplanned KRT initiation include a lower number of nephrology visits and under-coding of CKD (25). Mortality rates varied widely depending on the population studied and definitions used for late referral (usually within 1–6 months before KRT). Most of these studies did not differentiate CKD from AKI, or avoidable/unavoidable cases, or the presence of nephrological care (23, 24). In contrast, results of studies using early referrals (>180 days before KRT), or laboratory-confirmed CKD and nephrological care may be misleading. As shown, guideline-concordant patients had a higher risk of ESKD (HR: 7.65), CVDs (HR: 1.40), and mortality (HR: 1.58) compared to the guideline-discordant cohort, due to unjustified referrals of mild cases (26). Our nationwide study provides robust, real-world evidence for hard endpoints in a homogenous population of newly dialyzed chronic ESKD patients by comparing well-matched cohorts with undiagnosed and diagnosed CKD (using 2 month ICD-based definitions and P-EPO therapy to confirm prior nephrological care).

Consistent with literature data, KRT initiation occurred dominantly in males and older age groups; hemodialysis was the main modality (8, 10, 21). We found 10,810 ESKD patients starting KRT in 2014–2018 in Hungary, most of whom (67%) were diagnosed CKD patients. Kidney function tests increased with diagnosed CKD over 5 years pre-KRT, reflecting a better coding tendency with CKD progression (27). However, the frequency ranged from 77 to 97%, indicating that incomplete kidney function tests occurred even in patients with known CKD. About 50% of undiagnosed CKD patients had serum creatinine measured, yet their CKD was not formally recognized, despite their obviously declined kidney function in such a short term before requiring dialysis. Albuminuria testing clearly lagged behind, with rates between 5 and 14% in the undiagnosed group, and even among the identified CKD patients it remained suboptimal at 19–29%, consistent with earlier reports (16, 18, 28).

Key comorbidities were increasingly diagnosed toward KRT, but with higher rates for diagnosed CKD. Hypertension and T2DM were significantly more prevalent (92.9 and 46.7%) in the diagnosed CKD group compared to the undiagnosed group (59.6 and 26.2%, respectively). These prevalence data of undiagnosed CKD patients, together with the lower testing rates for CKD, could indicate their less access to healthcare and/or non-adherence to recommended medical management, but alternatively, it may also suggest under-recognition of CKD, even in high-risk individuals with hypertension and T2DM. Prevalence of hypertension, T2DM, CAD, and HF surpassed those published in the literature (1, 6, 16), but our data refer to pre-ESKD rather than to the entire CKD population. Prevalence of PKD (5.9%) and glomerulonephritis (11.2%) for only diagnosed CKD patients were consistent with global data (7).

Diagnosed CKD patients received more cardio-kidney-protective drugs that could afford decreased mortality, hospitalizations, and CVD morbidity (19, 29). The lower pre-KRT cancer incidence also suggests greater medical attention for diagnosed CKD patients. Cancer status was shown to determine all-cause mortality risk, but not ESKD progression and KRT uptake (30). Due to these clinical concerns, drugs and prior comorbidities (including cancers) were used for PS subclassification to avoid possible survival and morbidity advantages.

Undiagnosed CKD as a predictor of increased post-KRT cancer risk is a novel finding of this study. Increased new cancers and reduced cancer-free survival for undiagnosed CKD were consistent, and higher risk persisted at 12 months post-KRT. Unfortunately, we were unable to determine cancer-specific deaths.

Increasing laboratory testing of undiagnosed CKD patients was not accompanied by disease coding despite the presence of CKD-associated comorbidities, indicating low awareness of CKD due to under-reporting rather than insufficient screening of predisposed at-risk patients, as likewise in previous reports (6, 16–18). CKD was reported in 38% of undiagnosed CKD naive patients within 1 year before KRT initiation. Actually, they had CKD diagnosed within 2 months pre-KRT by our definition, therefore, these patients could be considered as late referrals, consistent with the general incidence of 20–50% for late referrals (23).

As mentioned earlier, only 50% of CKD naïve patients had serum creatinine tests over 5 years pre-index, despite their advanced CKD. Not only was CKD under-reported, but key comorbidities such as hypertension and diabetes were as well. These patients used fewer cardio-kidney-protective medications compared to CKD non-naive patients, while cancers showed a higher prevalence over 5 years pre-KRT. All these findings suggest that most undiagnosed CKD patients may have received fewer nephrological visits and less multidisciplinary medical care.

The high rates of mortality (27%), HHF (19%), and cancer (11.2%) observed in the CKD naïve group at 3 months, along with the short (3 months) median survival after dialysis initiation, were alarming. Moreover, these observed differences persisted after PS adjustment, supporting the validity and clinical relevance of our findings. Mortality among patients initiating dialysis is known to be highest during the first 90 days, with reported rates of approximately 27.5 deaths per 100 person-years, after which the annual mortality declines to around 20% (31, 32). Registry data showed that about one-third of deaths during the first dialysis year occur within the initial 90 days, particularly among older patients and those with CVDs, while pre-dialysis care lowers mortality (33). CVD accounts for 40–50% of deaths in this early period, with sudden cardiac death, arrhythmias, and HF being the most common causes; infection and malignancy are also important contributors (34). Early mortality rates are even higher in high-risk or late-referral cohorts, often exceeding 30%, particularly among patients who initiate dialysis without prior nephrology follow-up (23, 35). Therefore, although the magnitude of events observed in our CKD naïve group is striking, it is consistent with the well-described “early mortality” or “vulnerable period” following dialysis initiation.

The absence of a prior CKD diagnosis and pre-dialysis nephrology care in the CKD naïve group strongly suggests that many patients initiated dialysis in an unplanned manner (“crashing into dialysis”), without a managed transition to KRT. Such patients typically start treatment under unstable clinical conditions, with severe uremic symptoms, fluid overload, a high burden of untreated comorbidities (hypertension, diabetes, or CVD), and catheter-dependent vascular access. All of these factors increase the risk of early complications, including infection, HHF, and death (24, 25, 31). In addition, the lack of pre-ESKD management (no treatment of anemia, metabolic acidosis, or mineral bone disease) may further increase vulnerability during the dialysis transition (24, 25, 31). In this context, the short median survival of the CKD naïve patients suggests a very poor health status at the time of starting dialysis, possibly due to unrecognized/uncontrolled long-term comorbidities resulting from less medical attention, as their pre-index prevalence data suggested. The higher cancer rate also suggests these patients were extremely frail, making them more susceptible to a cancer diagnosis shortly after entering the healthcare system, or they might have suffered from latent malignancies worsened by severe uremia. Consistently, we found that higher early mortality was mainly driven by cancer, HHF, and stroke, and disproportionately affected the elderly population among CKD naïve patients. Taken together, the markedly elevated early event rates of the CKD naïve cohort likely reflect the clinical reality of the extremely late presentation of patients starting dialysis without prior CKD diagnosis or nephrology follow-up. Thus, while alarming, these findings highlight the substantial risks associated with delayed CKD recognition and the absence of structured pre-dialysis care.

Total healthcare costs increased toward KRT in both undiagnosed and diagnosed CKD cohorts, specifically in the last year, but more intensely post-KRT due to dialysis therapy, consistent with earlier reports (3, 4). According to Medicare claims data, CKD-related and total costs were higher for undiagnosed CKD (for stage 4–5 CKD or ESKD) compared to diagnosed cases (36). Conversely, we found that diagnosed CKD was associated with higher total costs both pre-KRT and post-KRT, except for the last year before KRT initiation.

However, total costs rose 7.5-fold in undiagnosed CKD and 4.5-fold in diagnosed CKD over the 5 years before dialysis, which was mainly related to higher hospitalization costs. Inpatient care spending for undiagnosed CKD contributed most (60%) to the total annualized expenditure at 1 year pre-KRT and remained significant over the 5 years post-KRT. Drug therapy costs were consistently higher for diagnosed CKD both before and after dialysis, reflecting more comprehensive long-term management, and possibly better adherence to polypharmacy among patients under regular visits (29).

Hospitalization is the major driver of CKD-related healthcare expenditure, followed by HF and atherosclerotic CVDs (6). CKD-associated AMI, stroke, and HF expenses are stable in Hungary (10). End-of-life encounters due to kidney failure accounted for longer hospital stays with higher costs compared to CVDs amongst CKD patients (37). Although admissions were of unknown causes, we can assume that inpatient care costs for undiagnosed CKD, based on the higher rates of deaths, HHF, and cancer, may have been associated with cardiorenal and cancer events in this study.

There are some limitations to this study. The NHIF registry contains no data on clinical parameters, thus kidney function, lipids, body mass index, smoking, vital signs, socioeconomic status, or indications for the first dialysis could not be obtained. It cannot be ruled out that these missing data could have influenced PS subclassification of patients, although matching of the two arms was perfect for 56 confounders. We made the two groups comparable by excluding higher-risk patients with a prior event, although this may undoubtedly lead to bias. Including kidney transplantation in the composite endpoint may complicate the interpretation; however, it accounted for a small proportion of events (4% in the CKD naive group and 14% in the CKD non-naive group) and thus did not drive the observed differences, which were mainly attributable to adverse clinical events. Cause-specific mortality data and CKD-related costs were also unavailable in the NHIF registry. As such, cancer types could not be evaluated.

In conclusion, one-third of newly dialyzed chronic ESKD patients in Hungary are undiagnosed for CKD and fail to receive nephrological care. We provided evidence that undiagnosed CKD among ESKD patients on KRT is a predictor of increased all-cause mortality and morbidity for CVDs (HHF in particular) and new cancers, and it reduces the likelihood of kidney transplantation. Undiagnosed CKD patients aged over 60 years require intensified medical attention during the first 18 months on dialysis due to the higher mortality risk from HHF, cancer, and stroke. We demonstrated that undiagnosed CKD requires higher hospitalization expenses, particularly in the year preceding dialysis. These findings underscore the need for early CKD diagnosis and appropriate interventions to reduce avoidable mortality and optimize healthcare resource utilization.

## Data Availability

The original contributions presented in the study are included in the article/Supplementary material, further inquiries can be directed to the corresponding author/s.
