# Supplementary material for: Comparison of risks and costs of undiagnosed and diagnosed chronic kidney disease patients after the initiation of kidney replacement therapy in subsequent years. A Hungarian nationwide study
Source: Front Med (Lausanne). 2026 Jul 21;13:1759417. doi: 10.3389/fmed.2026.1759417 (PMC13434849; doi:10.3389/fmed.2026.1759417)
Supplement: Supplementary file 1 [file Data_Sheet_1.PDF]

Supplementary material

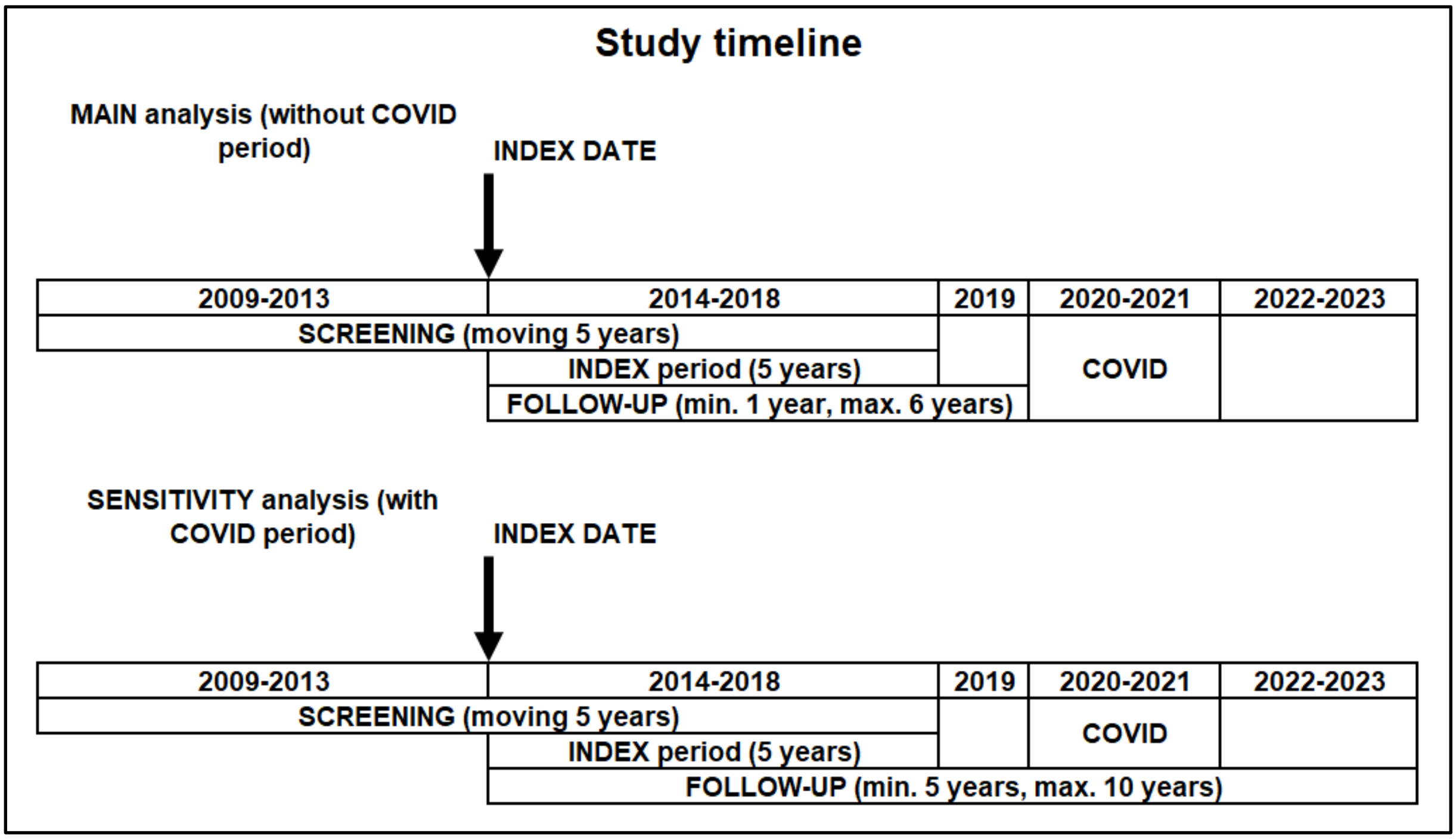

Figure S1. Overview of the observational study periods.
Data analysis was conducted between January 1, 2009, and September 30, 2023.
Administrative censoring was at 30th September, 2023.
Index period lasted between January 1, 2014 and December 31, 2018.
Index date refers to the first dialysis of patients with chronic end-stage kidney disease.
Screening period (5-years) was applied between January 1, 2009 and December 31.
Follow-up period started January 1, 2014 and ended December 31, 2019 (Main analysis), allowing a minimum of 1-year to a maximum of 6-years of post-index follow-up (without COVID outbreak in 2020-2021).
Sensitivity analysis continued until 2023 with a maximum of 116-months follow-up (including COVID).

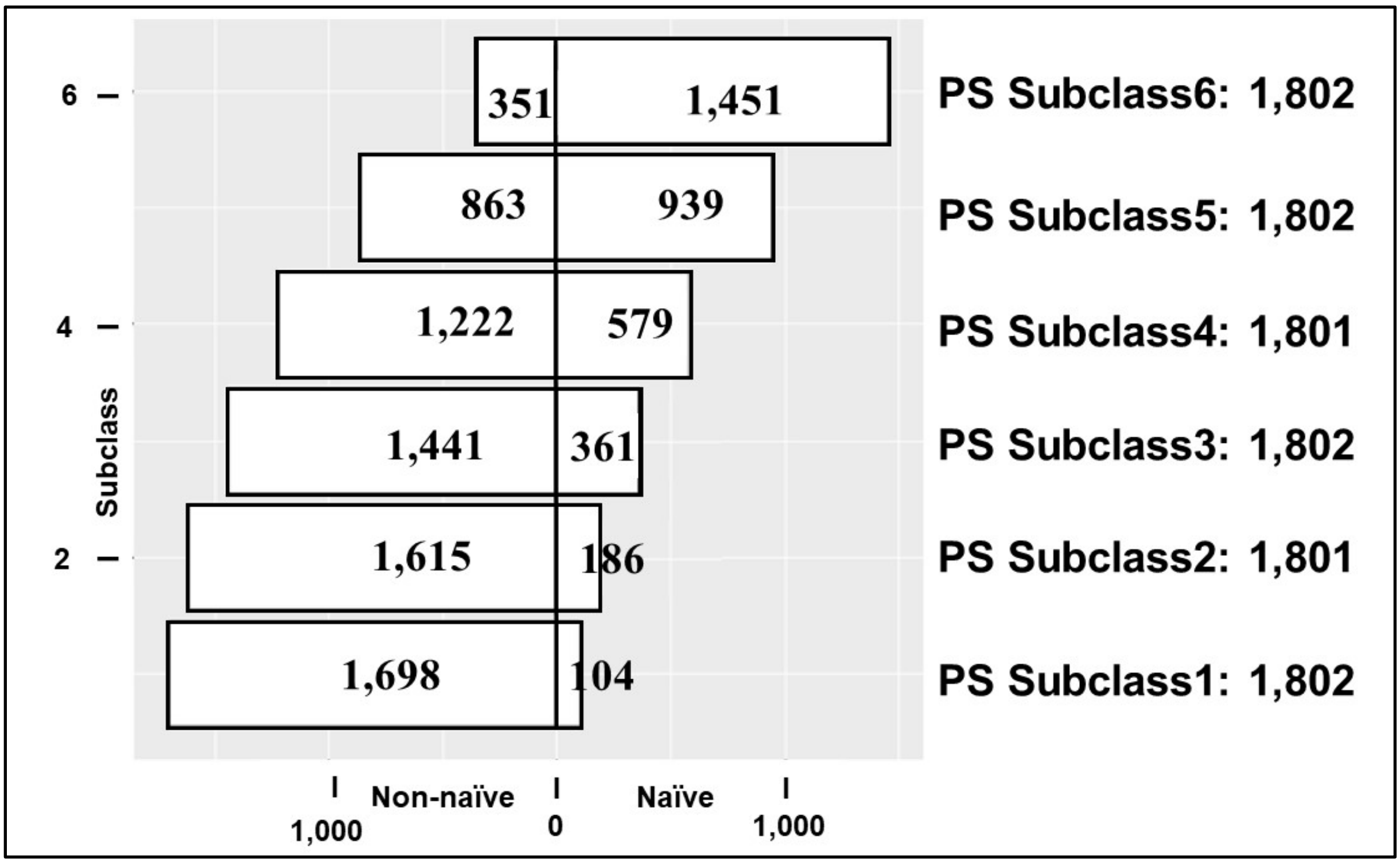

Figure S2. Propensity Score Subclassification (PS) method was used to create six equally sized clusters to compare the CKD naïve arm and the CKD non-naïve arm.
Distribution of selected 56 risk factors was well-balanced within each subclass.

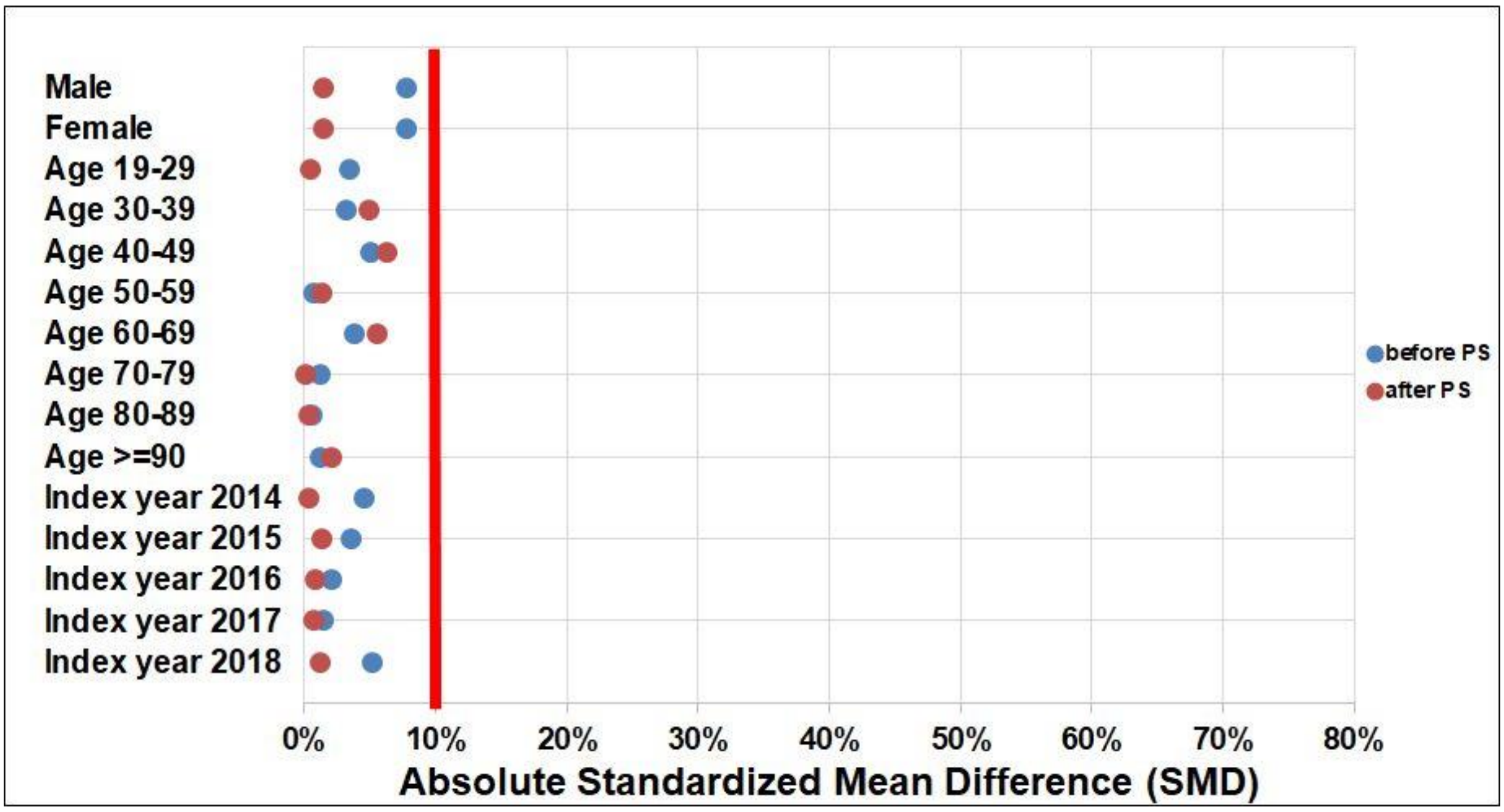

Figure S3. Standardized mean difference (SMD) values of demographic data (sex and age-groups) and index years (2014-2018) were all below the 10% cutoff before (blue) and after (red) Propensity Score Subclassification (PS), indicative of perfect matching of the two study arms.

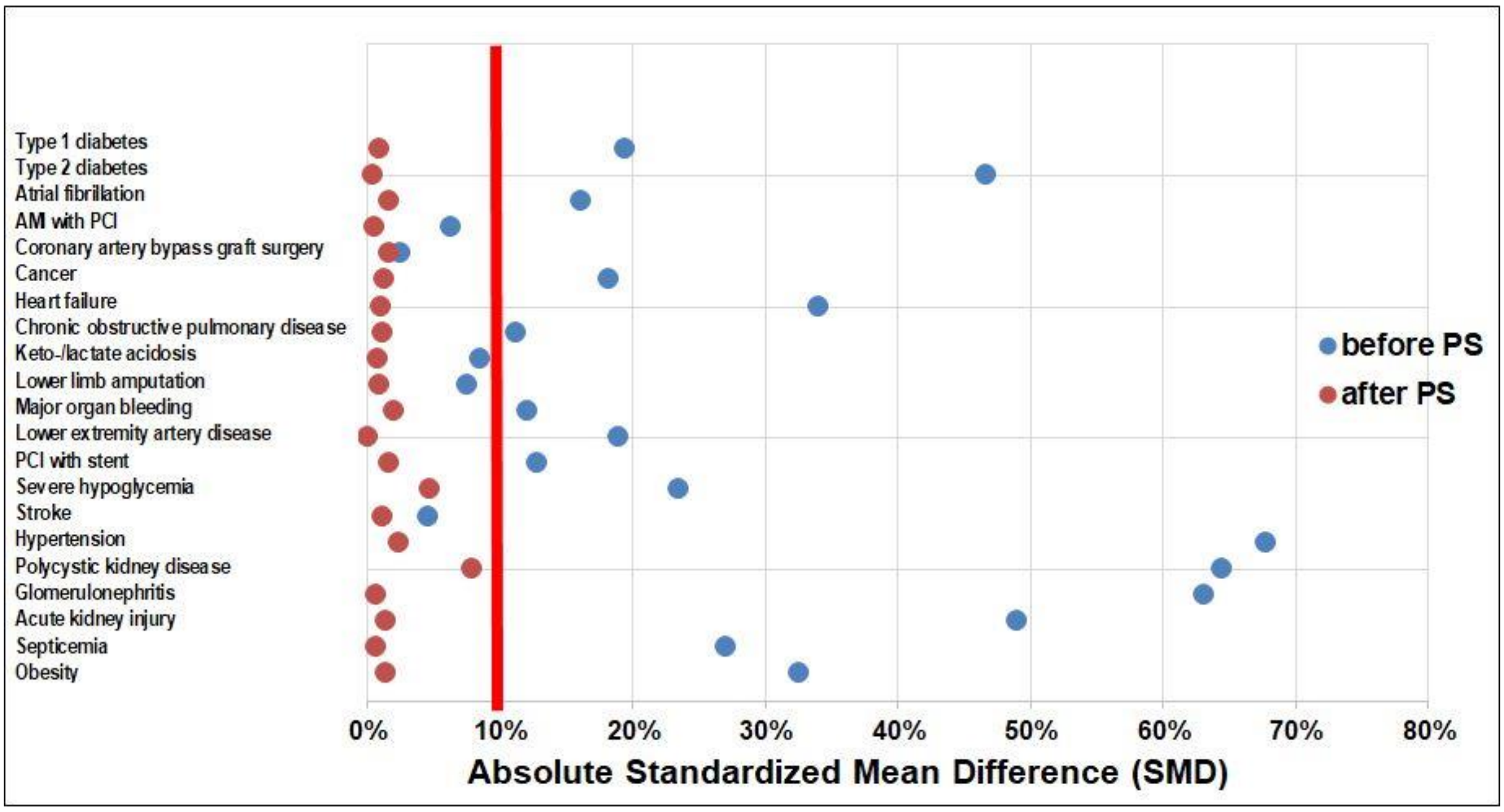

Figure S4. Baseline standardized mean difference (SMD) values for co-existing clinical disorders were very dissimilar before Propensity Score Subclassification (PS) (blue), all of which were within the 10% cutoff after PS (red), indicating the perfect matching of the two study arms.

(AMI: acute myocardial infarction; PCI: percutaneous coronary intervention)

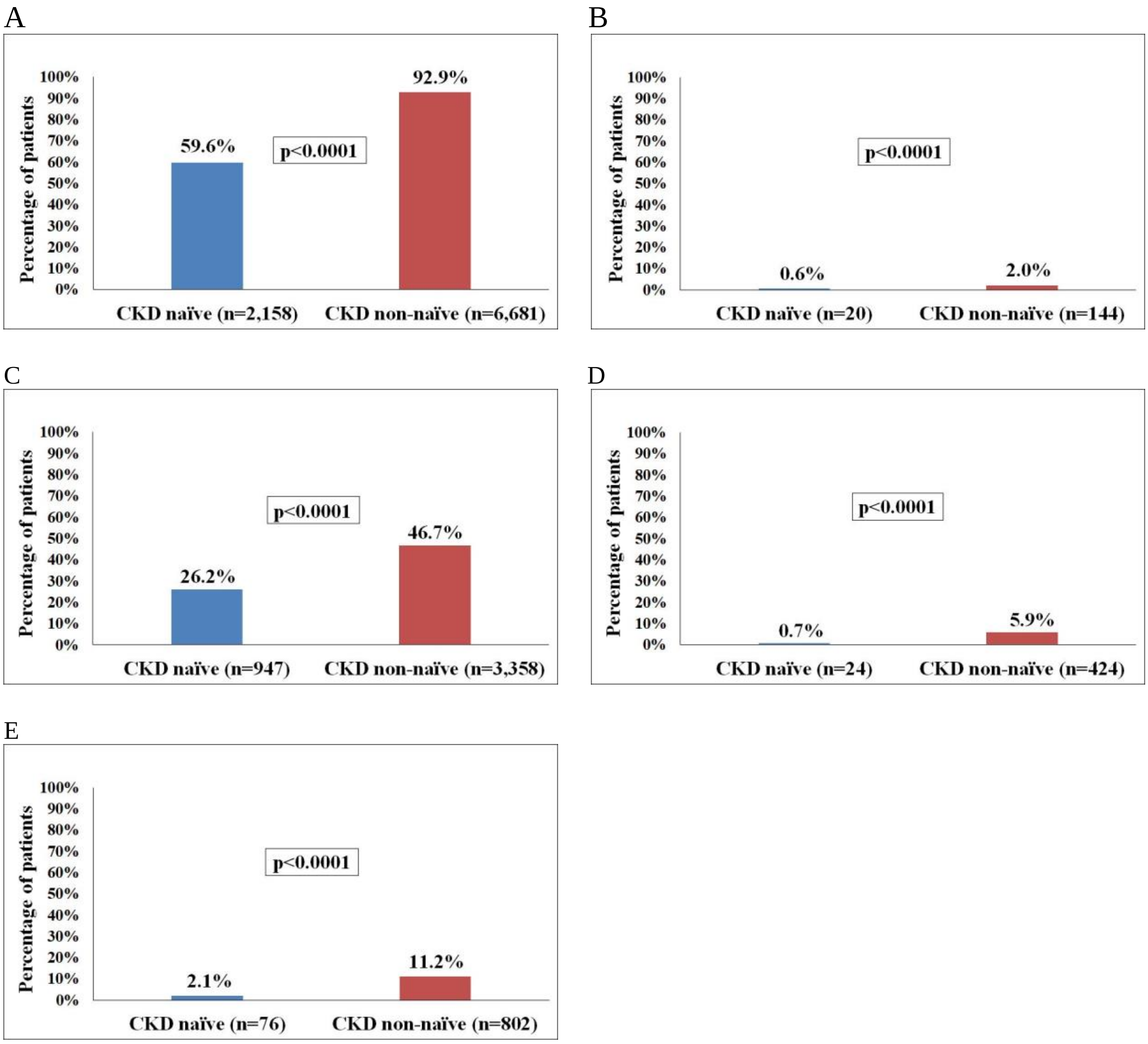

Figure S5. Prevalence of CKD-associated risk factors for (**A**) hypertension, (**B**) type 1 diabetes, and (**C**) type 2 diabetes, and underlying primary kidney diseases for (**D**) polycystic kidney disease and (**E**) glomerulonephritis in the comparison of the CKD naïve and CKD non-naïve groups in the preceding 5-years before the index date of the first dialysis.
$p<0.0001$ CKD naïve versus CKD non-naïve (Chi-squared test);

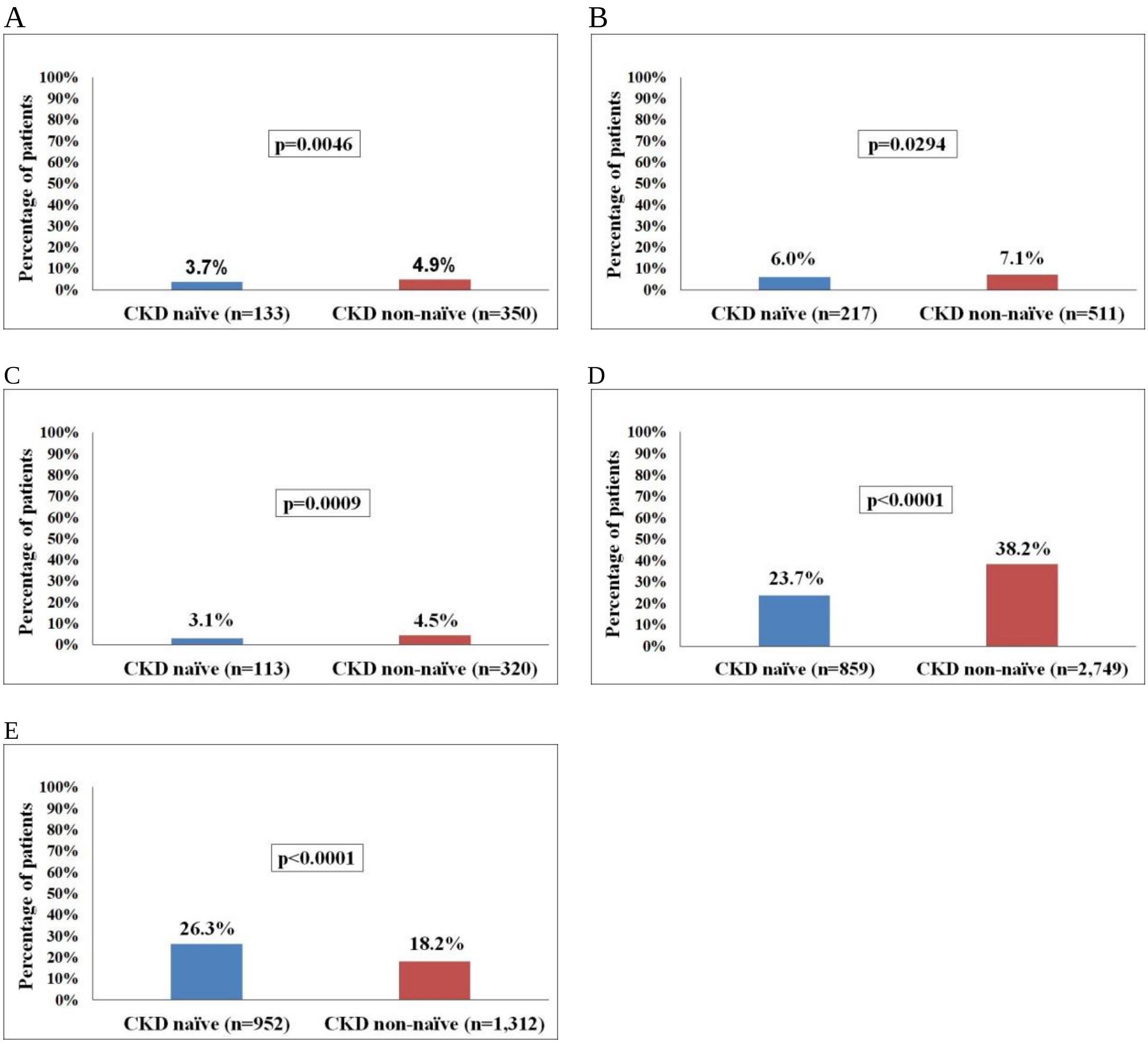

Figure S6. Prevalence of CKD-associated prior comorbidities for (**A**) acute myocardial infarction with percutaneous coronary intervention, (**B**) stroke, (**C**) lower limb amputation, (**D**) hospitalization for heart failure, and (**E**) cancer in the comparison of the CKD naïve and CKD non-naïve groups in the preceding 5-years before the index date of the first dialysis.
P values indicate CKD naïve versus CKD non-naïve (Chi-squared test);

A

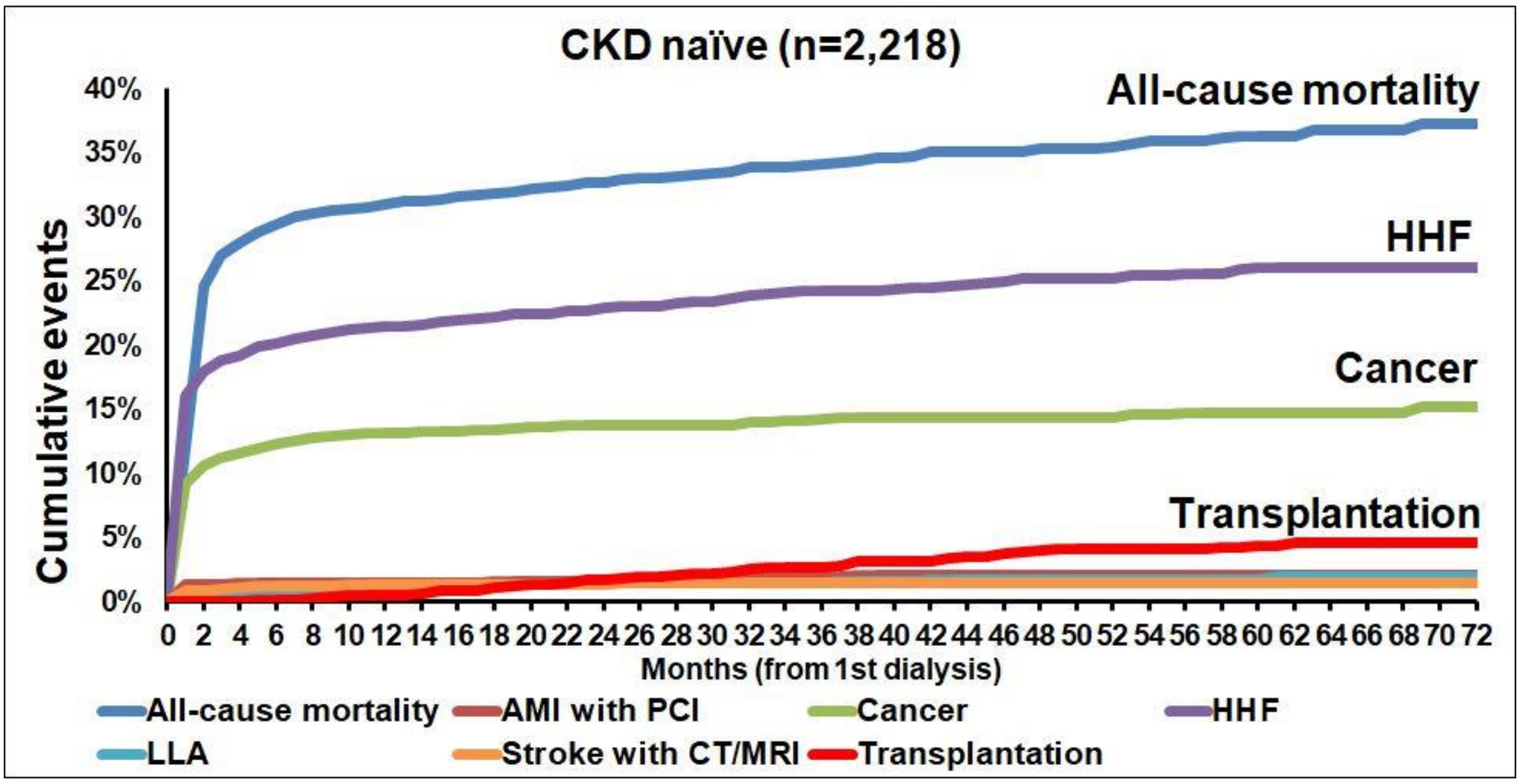

B

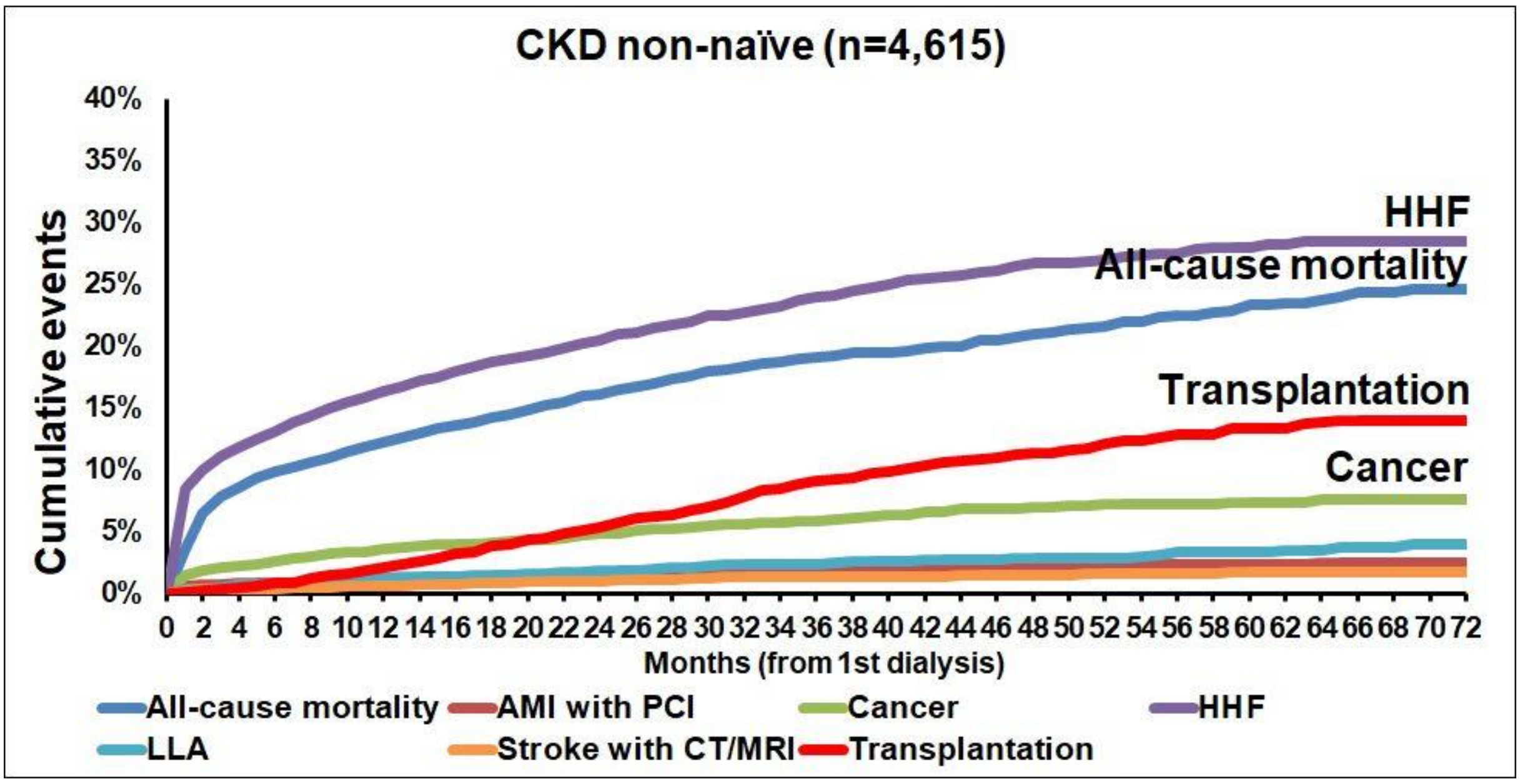

Figure S7. Cumulative incidence of primary endpoints from the time-to-first event analyses in the (**A**) CKD naïve arm (n=2,218) and (**B**) CKD non-naïve arm (n=4,615) during the 72-months follow-up after the first dialysis. Total number of events were n=1,815 in the CKD naïve arm and n=3,031 in the CKD non-naïve arm. Top 3 events were all-cause death, HHF (hospitalization for heart failure), and cancers in the CKD naive arm. Top 3 events were HHF, all-cause death, and kidney transplantation in the CKD non-naive arm.

Data are unadjusted for the different propensity score subclassification hazards (no hazards of the differences and statistics provided).

(AMI with PCI: acute myocardial infarction with percutaneous coronary intervention; LLA: lower limb amputation)

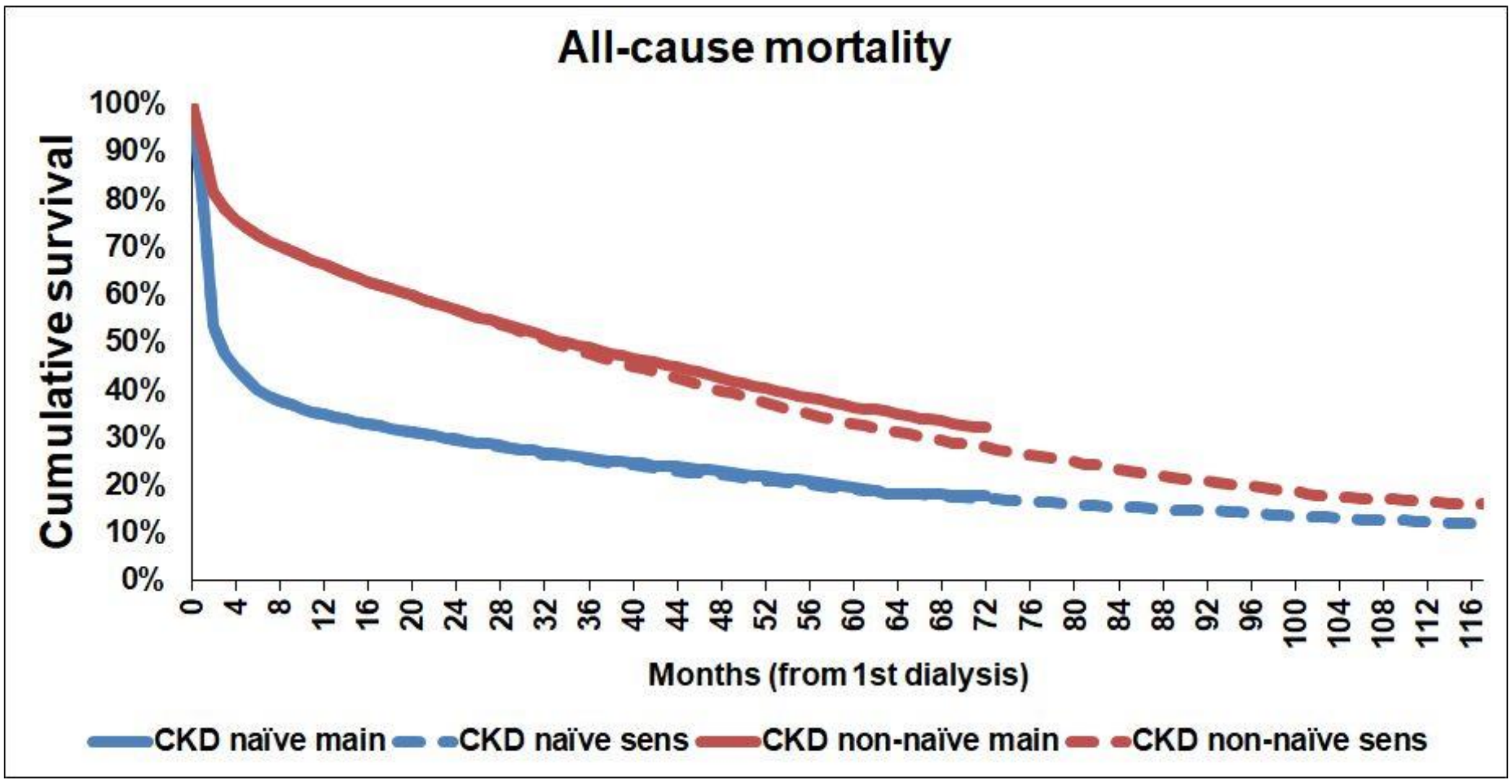

Figure S8. Kaplan-Meier survival curves from any cause of death in the CKD naïve arm (blue) and CKD non-naïve arm (red) during the main analysis of 72-months follow-up (continuous line) and the sensitivity analysis of 116-months follow-up (dotted line).

Data are unadjusted for the different propensity score subclassification hazards (no hazards of the differences and statistics provided).

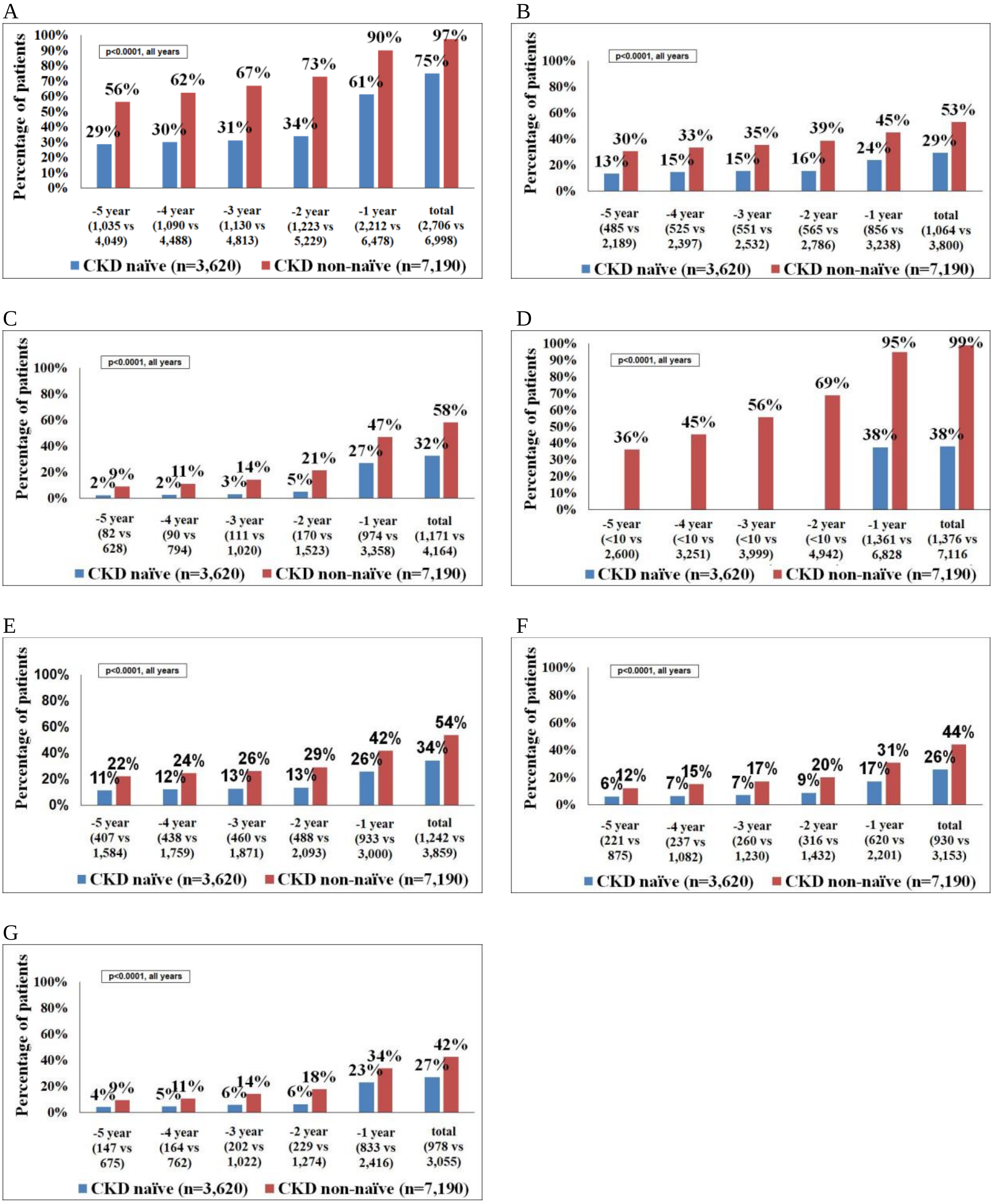

Figure S9. Annualized prevalence rates of (**A**) hypertension, (**B**) type 2 diabetes, (**C**) anemia, (**D**) chronic kidney disease, (**E**) coronary artery disease, (**F**) lower extremity artery disease, and (**G**) heart failure in the comparison of the CKD naïve and CKD non-naïve groups in the preceding 5-years before the first dialysis.

$p<0.0001$ CKD naïve versus CKD non-naïve (Chi-squared test);

A

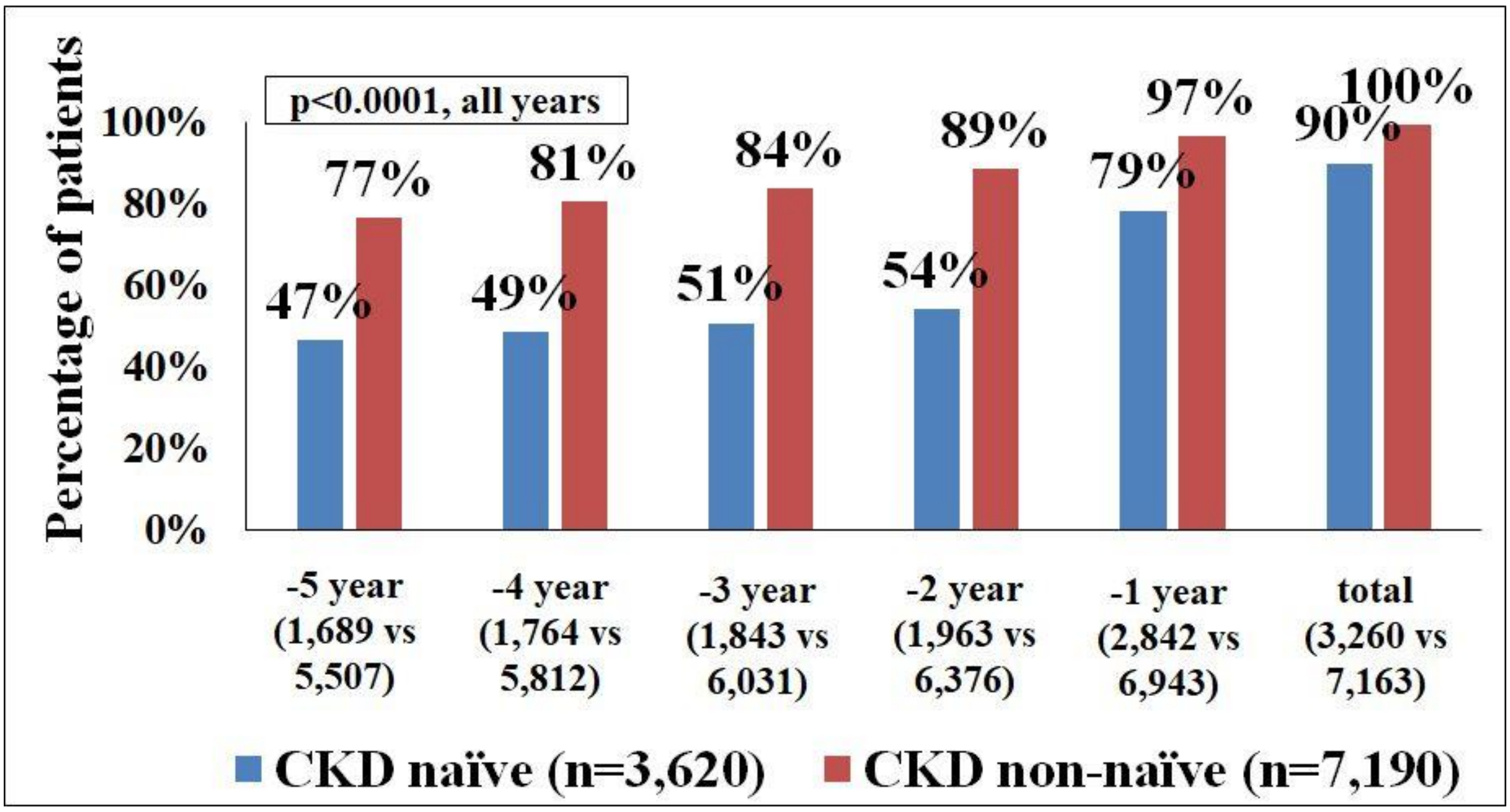

B

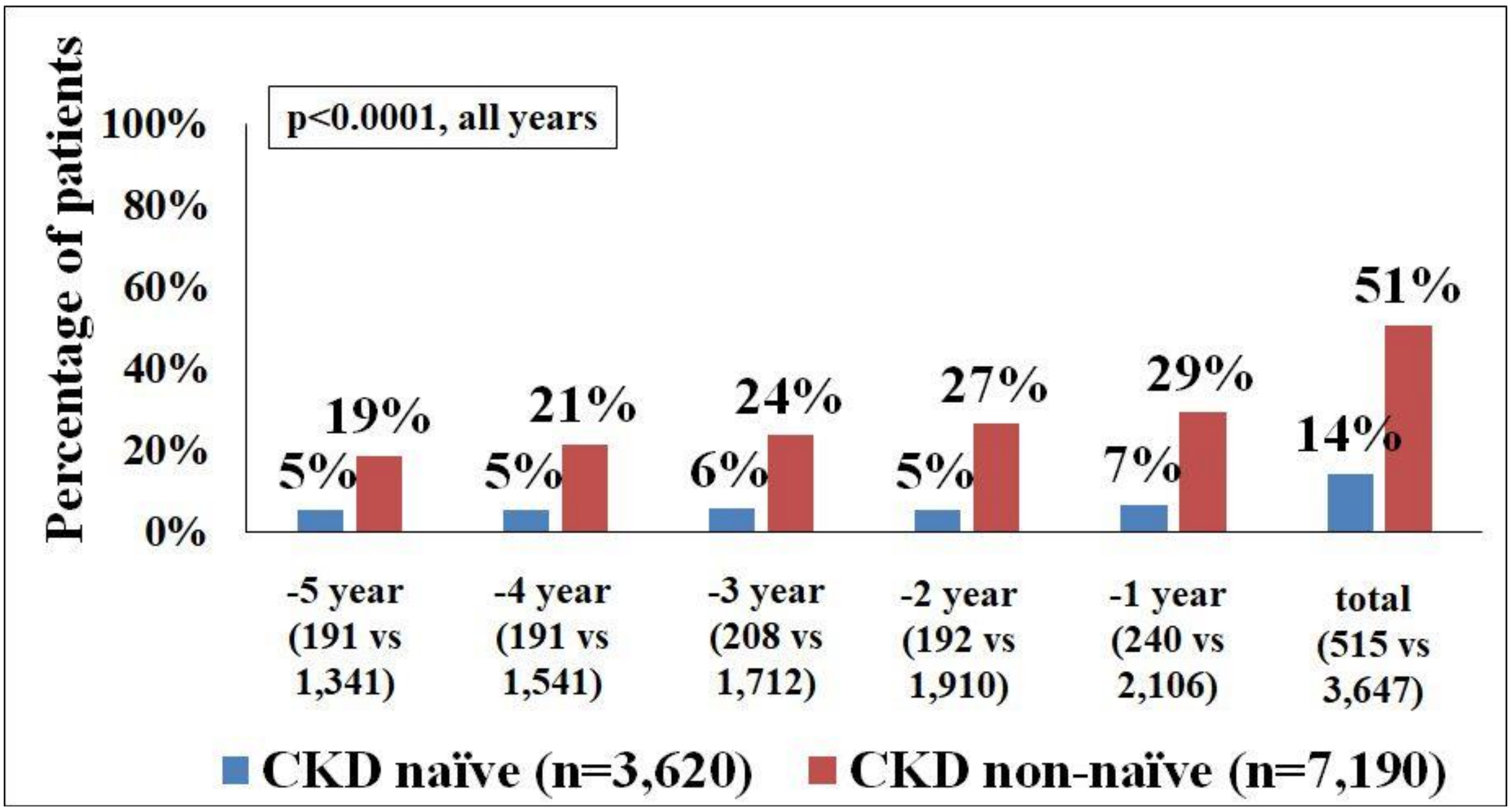

Figure S10. Annualized rates of laboratory testing for (**A**) serum creatinine and (**B**) albuminuria in the comparison of the CKD naïve and CKD non-naïve groups in the preceding 5-years before the first dialysis.
p<0.0001 CKD naïve versus CKD non-naïve (Chi-squared test);

A

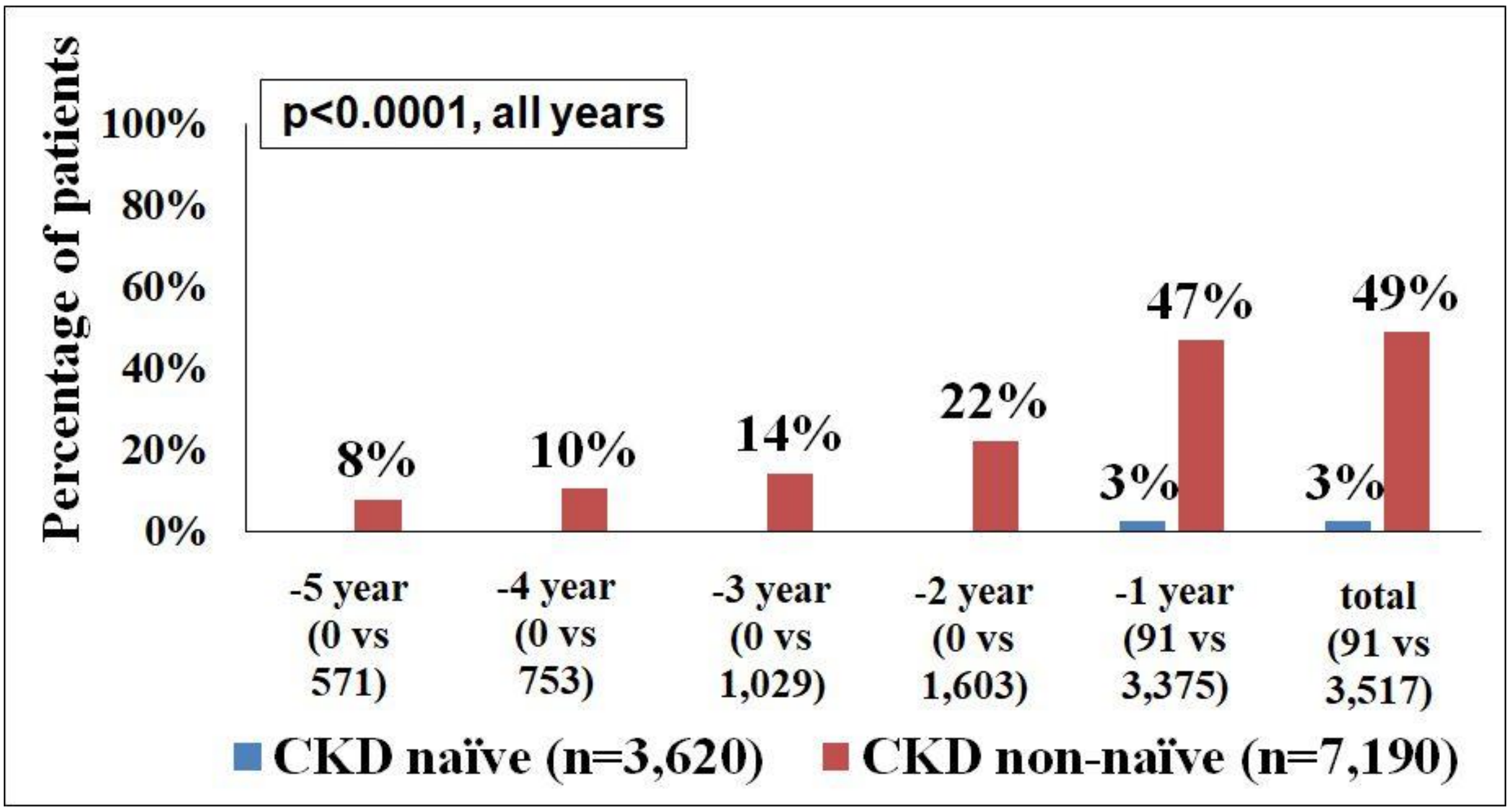

B

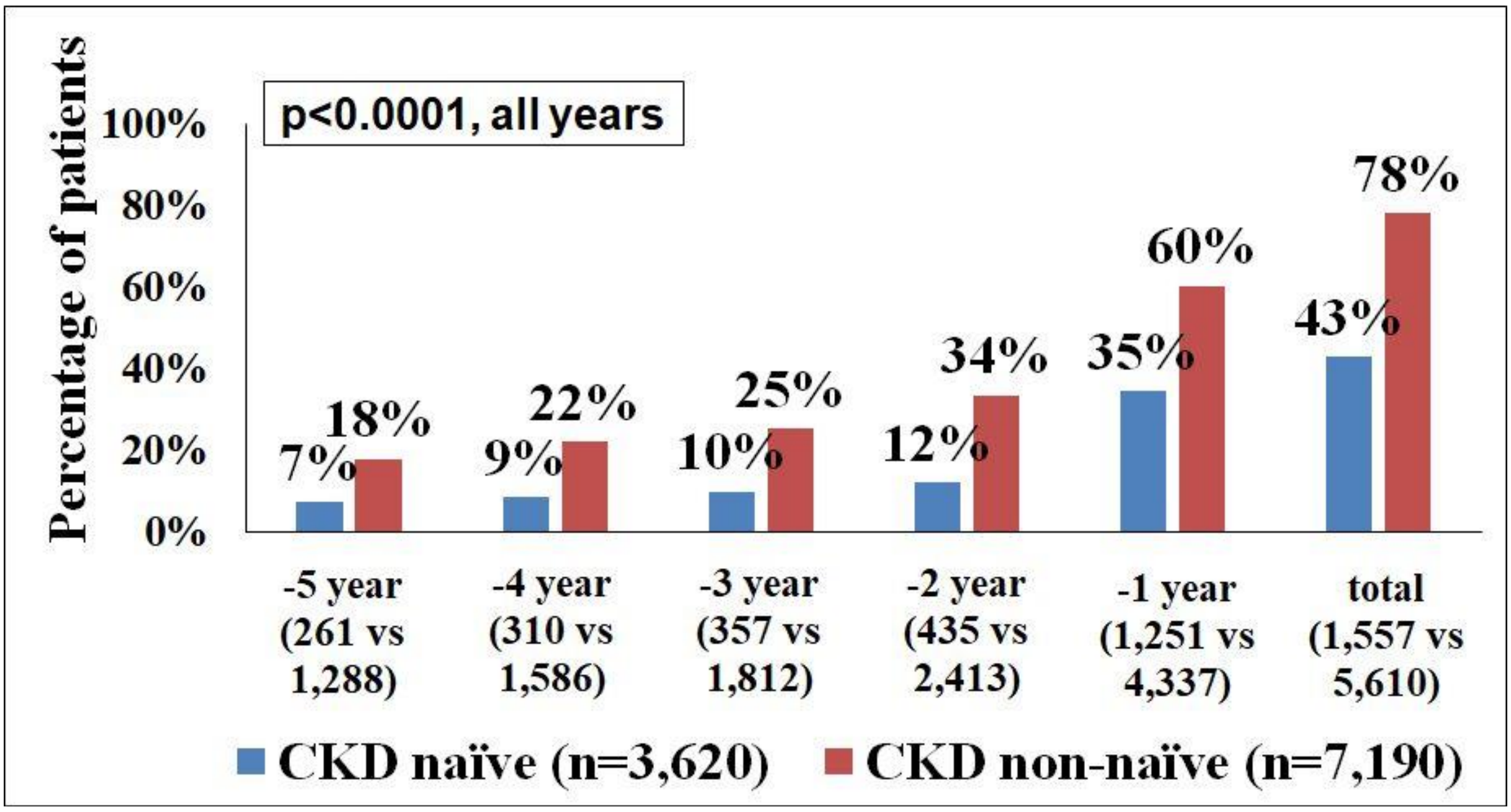

Figure S11. Annualized rates of drug therapy with (**A**) predialysis erythropoietin and (**B**) furosemide in the comparison of the CKD naïve and CKD non-naïve groups in the preceding 5-years before the first dialysis.

$p<0.0001$ CKD naïve versus CKD non-naïve (Chi-squared test);

Table S1. The number and percentage of CKD naïve and CKD non-naïve patients according to demographic data (sex and age-groups) and the observed index years (2014-2018) before (Unmatched) and after Propensity Score Subclassification (based on 6 subclasses).

	Unmatched			Propensity Score Subclassification												
				Subclass1		Subclass2		Subclass3		Subclass4		Subclass5		Subclass6		
	CKD naïve	CKD non-naïve	SMD	CKD naïve	CKD non-naïve	CKD naïve	CKD non-naïve	CKD naïve	CKD non-naïve	CKD naïve	CKD non-naïve	CKD naïve	CKD non-naïve	CKD naïve	CKD non-naïve	SMD
**Number of patients**	**3,620**	**7,190**		**104**	**1,698**	**186**	**1,615**	**361**	**1,441**	**579**	**1,222**	**939**	**863**	**1,451**	**351**	
Male n (%)	2,128 (58.8)	3,954 (55)	0.077	56 (53.8)	848 (49.9)	100 (53.8)	908 (56.2)	197 (54.6)	819 (56.8)	319 (55.1)	655 (53.6)	565 (60.2)	522 (60.5)	891 (61.4)	202 (57.5)	0.014
Age at index date																
19-29 years n (%)	68 (1.9)	102 (1.4)	0.034	<10 (2.9)	26 (1.5)	<10 (1.6)	19 (1.2)	<10 (0.3)	11 (0.8)	<10 (1)	17 (1.4)	16 (1.7)	15 (1.7)	39 (2.7)	14 (4)	0.005
30-39 years n (%)	117 (3.2)	273 (3.8)	0.032	<10 (4.8)	106 (6.2)	<10 (2.7)	41 (2.5)	<10 (1.1)	41 (2.8)	<10 (1.6)	44 (3.6)	25 (2.7)	23 (2.7)	69 (4.8)	18 (5.1)	0.049
40-49 years n (%)	255 (7)	598 (8.3)	0.050	10 (9.6)	192 (11.3)	<10 (4.8)	125 (7.7)	21 (5.8)	88 (6.1)	29 (5)	94 (7.7)	54 (5.8)	65 (7.5)	132 (9.1)	34 (9.7)	0.062
50-59 years n (%)	535 (14.8)	1,045 (14.5)	0.007	20 (19.2)	307 (18.1)	18 (9.7)	230 (14.2)	46 (12.7)	193 (13.4)	82 (14.2)	166 (13.6)	132 (14.1)	109 (12.6)	237 (16.3)	40 (11.4)	0.014
60-69 years n (%)	1,051 (29)	1,962 (27.3)	0.038	29 (27.9)	487 (28.7)	59 (31.7)	471 (29.2)	100 (27.7)	366 (25.4)	158 (27.3)	310 (25.4)	308 (32.8)	249 (28.9)	397 (27.4)	79 (22.5)	0.055
70-79 years n (%)	987 (27.3)	1,998 (27.8)	0.012	19 (18.3)	393 (23.1)	62 (33.3)	495 (30.7)	111 (30.7)	447 (31)	183 (31.6)	347 (28.4)	258 (27.5)	222 (25.7)	354 (24.4)	94 (26.8)	0.000
80-89 years n (%)	561 (15.5)	1,130 (15.7)	0.006	17 (16.3)	177 (10.4)	28 (15.1)	219 (13.6)	76 (21.1)	276 (19.2)	106 (18.3)	227 (18.6)	138 (14.7)	165 (19.1)	196 (13.5)	66 (18.8)	0.003
=>90 years n (%)	46 (1.3)	82 (1.1)	0.012	<10 (1)	10 (0.6)	<10 (1.1)	15 (0.9)	<10 (0.6)	19 (1.3)	<10 (1)	17 (1.4)	<10 (0.9)	15 (1.7)	27 (1.9)	<10 (1.7)	0.020
Index year																
2014 n (%)	771 (21.3)	1,397 (19.4)	0.046	18 (17.3)	301 (17.7)	35 (18.8)	307 (19)	67 (18.6)	281 (19.5)	125 (21.6)	248 (20.3)	207 (22)	175 (20.3)	319 (22)	85 (24.2)	0.003
2015 n (%)	790 (21.8)	1,463 (20.3)	0.036	21 (20.2)	319 (18.8)	44 (23.7)	319 (19.8)	66 (18.3)	310 (21.5)	113 (19.5)	248 (20.3)	228 (24.3)	188 (21.8)	318 (21.9)	79 (22.5)	0.013
2016 n (%)	697 (19.3)	1,441 (20)	0.020	24 (23.1)	344 (20.3)	36 (19.4)	345 (21.4)	65 (18)	280 (19.4)	114 (19.7)	251 (20.5)	170 (18.1)	168 (19.5)	288 (19.8)	53 (15.1)	0.008
2017 n (%)	713 (19.7)	1,458 (20.3)	0.015	20 (19.2)	372 (21.9)	38 (20.4)	324 (20.1)	80 (22.2)	276 (19.2)	108 (18.7)	235 (19.2)	175 (18.6)	184 (21.3)	292 (20.1)	67 (19.1)	0.006
2018 n (%)	649 (17.9)	1,431 (19.9)	0.051	21 (20.2)	362 (21.3)	33 (17.7)	320 (19.8)	83 (23)	294 (20.4)	119 (20.6)	240 (19.6)	159 (16.9)	148 (17.1)	234 (16.1)	67 (19.1)	0.012

Table S2. The number and percentage of CKD naïve and CKD non-naïve patients according to co-existing clinical disorders before (Unmatched) and after Propensity Score Subclassification (based on 6 subclasses).

	Unmatched			Propensity-Score Subclassification												
				Subclass1		Subclass2		Subclass3		Subclass4		Subclass5		Subclass6		
	CKD naïve	CKD non-naïve	SMD	CKD naïve	CKD non-naïve	CKD naïve	CKD non-naïve	CKD naïve	CKD non-naïve	CKD naïve	CKD non-naïve	CKD naïve	CKD non-naïve	CKD naïve	CKD non-naïve	SMD
Number of patients	3,620	7,190		104	1,698	186	1,615	361	1,441	579	1,222	939	863	1,451	351	
Type 1 diabetes mellitus n (%)	20 (0.6)	144 (2)	0.196	<10 (5.8)	75 (4.4)	<10 (1.1)	47 (2.9)	<10 (0.3)	15 (1)	<10 (0.3)	<10 (0.3)	<10 (0.9)	<10 (0.3)	<10 (0.1)	0 (0)	0.010
Type 2 diabetes mellitus n (%)	947 (26.2)	3,358 (46.7)	0.467	57 (54.8)	951 (56)	135 (72.6)	1,107 (68.5)	186 (51.5)	773 (53.6)	130 (22.5)	247 (20.2)	249 (26.5)	213 (24.7)	190 (13.1)	67 (19.1)	0.004
Atrial fibrillation n (%)	396 (10.9)	1149 (16)	0.162	21 (20.2)	289 (17)	41 (22)	293 (18.1)	79 (21.9)	245 (17)	74 (12.8)	190 (15.5)	105 (11.2)	92 (10.7)	76 (5.2)	40 (11.4)	0.017
AMI with PCI n (%)	133 (3.7)	350 (4.9)	0.063	<10 (3.8)	84 (4.9)	<10 (4.8)	95 (5.9)	26 (7.2)	70 (4.9)	31 (5.4)	56 (4.6)	29 (3.1)	34 (3.9)	34 (2.3)	11 (3.1)	0.006
CABG surgery n (%)	41 (1.1)	101 (1.4)	0.026	0 (0)	15 (0.9)	<10 (4.3)	27 (1.7)	<10 (2.5)	25 (1.7)	<10 (0.9)	15 (1.2)	11 (1.2)	16 (1.9)	<10 (0.6)	<10 (0.9)	0.017
Cancer n (%)	952 (26.3)	1,312 (18.2)	0.183	10 (9.6)	166 (9.8)	32 (17.2)	210 (13)	71 (19.7)	276 (19.2)	119 (20.6)	270 (22.1)	356 (37.9)	287 (33.3)	364 (25.1)	103 (29.3)	0.014
Heart failure n (%)	859 (23.7)	2,749 (38.2)	0.341	50 (48.1)	760 (44.8)	98 (52.7)	772 (47.8)	161 (44.6)	569 (39.5)	173 (29.9)	375 (30.7)	205 (21.8)	204 (23.6)	172 (11.9)	69 (19.7)	0.011
COPD n (%)	412 (11.4)	1,077 (15)	0.113	20 (19.2)	248 (14.6)	29 (15.6)	281 (17.4)	67 (18.6)	217 (15.1)	94 (16.2)	171 (14)	118 (12.6)	125 (14.5)	84 (5.8)	35 (10)	0.012
Keto-/lactic acidosis n (%)	<10 (0.2)	45 (0.6)	0.086	<10 (1.9)	20 (1.2)	<10 (0.5)	11 (0.7)	<10 (0.6)	<10 (0.6)	<10 (0.2)	<10 (0.1)	<10 (0.1)	<10 (0.5)	<10 (0.1)	0 (0)	0.009
Lower limb amputation n (%)	113 (3.1)	320 (4.5)	0.076	<10 (3.8)	85 (5)	<10 (4.3)	79 (4.9)	21 (5.8)	84 (5.8)	15 (2.6)	43 (3.5)	36 (3.8)	24 (2.8)	29 (2)	<10 (1.4)	0.009
Major bleeding n (%)	219 (6)	644 (9)	0.122	13 (12.5)	213 (12.5)	11 (5.9)	153 (9.5)	32 (8.9)	127 (8.8)	48 (8.3)	85 (7)	52 (5.5)	46 (5.3)	63 (4.3)	20 (5.7)	0.021
Lower extremity artery disease n (%)	265 (7.3)	882 (12.3)	0.190	16 (15.4)	266 (15.7)	29 (15.6)	224 (13.9)	53 (14.7)	208 (14.4)	47 (8.1)	118 (9.7)	60 (6.4)	50 (5.8)	60 (4.1)	16 (4.6)	0.002
PCI with stenting n (%)	180 (5)	559 (7.8)	0.129	<10 (6.7)	152 (9)	16 (8.6)	163 (10.1)	32 (8.9)	117 (8.1)	44 (7.6)	67 (5.5)	46 (4.9)	48 (5.6)	35 (2.4)	12 (3.4)	0.017
Severe hypoglycemia n (%)	<10 (0.2)	102 (1.4)	0.235	<10 (1)	81 (4.8)	<10 (1.6)	12 (0.7)	<10 (0.8)	<10 (0.3)	0 (0)	<10 (0.3)	<10 (0.1)	0 (0)	<10 (0.1)	0 (0)	0.048
Stroke n (%)	217 (6)	511 (7.1)	0.047	<10 (6.7)	112 (6.6)	18 (9.7)	108 (6.7)	29 (8)	112 (7.8)	45 (7.8)	86 (7)	79 (8.4)	77 (8.9)	39 (2.7)	16 (4.6)	0.012
Hypertension n (%)	2,158 (59.6)	6,681 (92.9)	0.679	104 (100)	1,692 (99.6)	184 (98.9)	1,597 (98.9)	353 (97.8)	1,417 (98.3)	562 (97.1)	1,166 (95.4)	824 (87.8)	749 (86.8)	131 (9)	60 (17.1)	0.024
Polycystic kidney disease n (%)	24 (0.7)	424 (5.9)	0.645	15 (14.4)	374 (22)	<10 (0.5)	29 (1.8)	<10 (1.7)	13 (0.9)	<10 (0.3)	<10 (0.7)	0 (0)	0 (0)	0 (0)	0 (0)	0.080
Glomerulonephritis n (%)	76 (2.1)	802 (11.2)	0.632	35 (33.7)	514 (30.3)	22 (11.8)	219 (13.6)	<10 (1.4)	28 (1.9)	<10 (0.9)	22 (1.8)	<10 (1)	19 (2.2)	0 (0)	0 (0)	0.007
Acute kidney injury n (%)	88 (2.4)	718 (10)	0.491	26 (25)	430 (25.3)	20 (10.8)	165 (10.2)	22 (6.1)	73 (5.1)	<10 (0.9)	19 (1.6)	10 (1.1)	28 (3.2)	<10 (0.3)	<10 (0.9)	0.015
Septicemia n (%)	64 (1.8)	384 (5.3)	0.271	13 (12.5)	208 (12.2)	12 (6.5)	90 (5.6)	11 (3)	41 (2.8)	14 (2.4)	24 (2)	<10 (0.7)	13 (1.5)	<10 (0.5)	<10 (2.3)	0.007
Obesity n (%)	251 (6.9)	1,094 (15.2)	0.326	22 (21.2)	368 (21.7)	37 (19.9)	345 (21.4)	45 (12.5)	217 (15.1)	58 (10)	112 (9.2)	62 (6.6)	43 (5)	27 (1.9)	<10 (2.6)	0.015

(AMI: acute myocardial infarction; CABG: coronary artery bypass graft surgery; COPD: chronic obstructive pulmonary disease; PCI: percutaneous coronary intervention)

Table S3. The number and percentage of CKD naïve and CKD non-naïve patients according to medications before (Unmatched) and after Propensity Score Subclassification (based on 6 subclasses).

	Unmatched			Propensity Score Subclassification												
				Subclass1		Subclass2		Subclass3		Subclass4		Subclass5		Subclass6		
	CKD naïve	CKD non-naïve	SMD	CKD naïve	CKD non-naïve	CKD naïve	CKD non-naïve	CKD naïve	CKD non-naïve	CKD naïve	CKD non-naïve	CKD naïve	CKD non-naïve	CKD naïve	CKD non-naïve	SMD
Number of patients	3,620	7,190		104	1,698	186	1,615	361	1,441	579	1,222	939	863	1,451	351	
Metformin n (%)	364 (10.1)	292 (4.1)	0.199	0 (0)	<10 (0.4)	<10 (3.2)	19 (1.2)	<10 (1.9)	37 (2.6)	40 (6.9)	51 (4.2)	147 (15.7)	125 (14.5)	164 (11.3)	54 (15.4)	0.006
Sulphonylurea n (%)	382 (10.6)	932 (13)	0.078	20 (19.2)	190 (11.2)	41 (22)	251 (15.5)	52 (14.4)	236 (16.4)	61 (10.5)	108 (8.8)	104 (11.1)	107 (12.4)	104 (7.2)	40 (11.4)	0.045
DPP-4i n (%)	109 (3)	189 (2.6)	0.022	<10 (1.9)	24 (1.4)	<10 (2.2)	26 (1.6)	<10 (2.2)	42 (2.9)	32 (5.5)	47 (3.8)	36 (3.8)	35 (4.1)	27 (1.9)	15 (4.3)	0.006
SGLT2i n (%)	18 (0.5)	11 (0.2)	0.049	0 (0)	<10 (0.2)	0 (0)	0 (0)	<10 (0.3)	0 (0)	<10 (0.3)	<10 (0.2)	<10 (0.2)	<10 (0.3)	13 (0.9)	<10 (0.9)	0.006
GLP-1 RA n (%)	<10 (0.2)	<10 (0.1)	0.010	0 (0)	<10 (0.1)	0 (0)	<10 (0.1)	0 (0)	<10 (0.2)	<10 (0.3)	<10 (0.1)	<10 (0.1)	<10 (0.2)	<10 (0.2)	0 (0)	0.002
Metiglinides n (%)	0 (0)	<10 (0)	0.020	0 (0)	<10 (0.1)	0 (0)	0 (0)	0 (0)	0 (0)	0 (0)	0 (0)	0 (0)	0 (0)	0 (0)	0 (0)	0.017
Glitazones n (%)	<10 (0.1)	<10 (0.1)	0.006	<10 (1)	<10 (0.1)	0 (0)	<10 (0.1)	0 (0)	0 (0)	0 (0)	<10 (0.1)	0 (0)	<10 (0.1)	<10 (0.1)	0 (0)	0.044
Acarbose n (%)	<10 (0.2)	55 (0.8)	0.147	0 (0)	34 (2)	<10 (2.2)	12 (0.7)	0 (0)	<10 (0.5)	<10 (0.2)	<10 (0.1)	<10 (0.1)	<10 (0.1)	0 (0)	0 (0)	0.025
Short acting insulin (human/analog) n (%)	215 (5.9)	1,100 (15.3)	0.396	26 (25)	406 (23.9)	44 (23.7)	372 (23)	50 (13.9)	202 (14)	33 (5.7)	60 (4.9)	51 (5.4)	45 (5.2)	11 (0.8)	15 (4.3)	0.005
Intermediate insulin (human basal) n (%)	145 (4)	729 (10.1)	0.313	20 (19.2)	270 (15.9)	35 (18.8)	239 (14.8)	31 (8.6)	132 (9.2)	18 (3.1)	43 (3.5)	33 (3.5)	33 (3.8)	<10 (0.6)	12 (3.4)	0.021
Long-acting insulin (analog basal) n (%)	85 (2.3)	327 (4.5)	0.145	<10 (4.8)	87 (5.1)	19 (10.2)	112 (6.9)	17 (4.7)	83 (5.8)	<10 (1)	24 (2)	22 (2.3)	17 (2)	16 (1.1)	<10 (1.1)	0.012
Premix insulin (human/analog) n (%)	99 (2.7)	530 (7.4)	0.284	11 (10.6)	206 (12.1)	19 (10.2)	192 (11.9)	22 (6.1)	80 (5.6)	17 (2.9)	31 (2.5)	24 (2.6)	17 (2)	<10 (0.4)	<10 (1.1)	0.019
Low dose aspirin n (%)	684 (18.9)	1,692 (23.5)	0.118	31 (29.8)	397 (23.4)	46 (24.7)	418 (25.9)	98 (27.1)	359 (24.9)	134 (23.1)	281 (23)	192 (20.4)	178 (20.6)	183 (12.6)	59 (16.8)	0.013
Statin n (%)	1,003 (27.7)	2,999 (41.7)	0.313	49 (47.1)	893 (52.6)	89 (47.8)	755 (46.7)	156 (43.2)	597 (41.4)	215 (37.1)	454 (37.2)	271 (28.9)	224 (26)	223 (15.4)	76 (21.7)	0.021
ACEi n (%)	1,823 (50.4)	3,575 (49.7)	0.013	52 (50)	714 (42)	99 (53.2)	806 (49.9)	193 (53.5)	737 (51.1)	298 (51.5)	622 (50.9)	601 (64)	535 (62)	580 (40)	161 (45.9)	0.034
ARB n (%)	671 (18.5)	1,969 (27.4)	0.228	32 (30.8)	578 (34)	59 (31.7)	487 (30.2)	108 (29.9)	397 (27.6)	140 (24.2)	280 (22.9)	179 (19.1)	178 (20.6)	153 (10.5)	49 (14)	0.012
Dihydropyridine CCB n (%)	743 (20.5)	2,633 (36.6)	0.399	53 (51)	845 (49.8)	77 (41.4)	677 (41.9)	132 (36.6)	551 (38.2)	205 (35.4)	362 (29.6)	135 (14.4)	152 (17.6)	141 (9.7)	46 (13.1)	0.007
Loop diuretics n (%)	57 (1.6)	211 (2.9)	0.109	<10 (3.8)	81 (4.8)	<10 (2.7)	46 (2.8)	12 (3.3)	42 (2.9)	14 (2.4)	25 (2)	<10 (1)	10 (1.2)	13 (0.9)	<10 (2)	0.018
Beta blockers n (%)	1,411 (39)	3,487 (48.5)	0.195	53 (51)	894 (52.7)	104 (55.9)	822 (50.9)	189 (52.4)	752 (52.2)	271 (46.8)	527 (43.1)	405 (43.1)	362 (41.9)	389 (26.8)	130 (37)	0.006
Non-dihydropyridine CCB n (%)	33 (0.9)	137 (1.9)	0.105	<10 (3.8)	51 (3)	<10 (3.8)	35 (2.2)	<10 (1.4)	27 (1.9)	<10 (0.7)	16 (1.3)	<10 (0.7)	<10 (0.8)	<10 (0.4)	<10 (0.3)	0.020
Aldosterone antagonist n (%)	553 (15.3)	1,067 (14.8)	0.012	19 (18.3)	213 (12.5)	31 (16.7)	231 (14.3)	60 (16.6)	225 (15.6)	88 (15.2)	168 (13.7)	185 (19.7)	173 (20)	170 (11.7)	57 (16.2)	0.026
Digitoxin n (%)	53 (1.5)	248 (3.4)	0.165	<10 (5.8)	94 (5.5)	<10 (3.8)	75 (4.6)	14 (3.9)	40 (2.8)	12 (2.1)	27 (2.2)	10 (1.1)	<10 (1)	<10 (0.3)	<10 (0.9)	0.003
Digoxin n (%)	121 (3.3)	144 (2)	0.075	<10 (1.9)	<10 (0.5)	<10 (1.1)	18 (1.1)	13 (3.6)	36 (2.5)	15 (2.6)	26 (2.1)	41 (4.4)	35 (4.1)	48 (3.3)	20 (5.7)	0.009
Amiodarone n (%)	90 (2.5)	216 (3)	0.033	<10 (1.9)	39 (2.3)	12 (6.5)	53 (3.3)	10 (2.8)	52 (3.6)	22 (3.8)	45 (3.7)	21 (2.2)	16 (1.9)	23 (1.6)	11 (3.1)	0.009
Warfarin n (%)	98 (2.7)	225 (3.1)	0.026	<10 (2.9)	48 (2.8)	<10 (3.2)	60 (3.7)	<10 (2.5)	39 (2.7)	23 (4)	42 (3.4)	30 (3.2)	31 (3.6)	27 (1.9)	<10 (1.4)	0.001
P2Y12 receptor antagonists n (%)	285 (7.9)	798 (11.1)	0.120	13 (12.5)	198 (11.7)	35 (18.8)	202 (12.5)	47 (13)	168 (11.7)	65 (11.2)	112 (9.2)	75 (8)	94 (10.9)	50 (3.4)	24 (6.8)	0.024
Factor Xa inhibitor n (%	34 (0.9)	67 (0.9)	0.001	0 (0)	13 (0.8)	<10 (1.1)	10 (0.6)	<10 (1.7)	17 (1.2)	<10 (0.7)	12 (1)	14 (1.5)	11 (1.3)	<10 (0.6)	<10 (1.1)	0.008
Direct thrombin inhibitor n (%)	19 (0.5)	23 (0.3)	0.028	<10 (1)	<10 (0.1)	0 (0)	<10 (0.2)	<10 (0.3)	<10 (0.3)	<10 (0.5)	<10 (0.5)	10 (1.1)	<10 (0.5)	<10 (0.3)	<10 (1.4)	0.005
Other antiplatelets n (%)	55 (1.5)	113 (1.6)	0.004	<10 (1)	15 (0.9)	<10 (2.2)	21 (1.3)	<10 (2.5)	37 (2.6)	<10 (1)	23 (1.9)	17 (1.8)	14 (1.6)	18 (1.2)	<10 (0.9)	0.008
Corticosteroids n (%)	269 (7.4)	681 (9.5)	0.078	16 (15.4)	207 (12.2)	17 (9.1)	138 (8.5)	33 (9.1)	137 (9.5)	47 (8.1)	110 (9)	74 (7.9)	62 (7.2)	82 (5.7)	27 (7.7)	0.007
Weight loss medication n (%)	0 (0)	0 (0)	0.000	0 (0)	0 (0)	0 (0)	0 (0)	0 (0)	0 (0)	0 (0)	0 (0)	0 (0)	0 (0)	0 (0)	0 (0)	0.000
Antibiotics n (%)	1,700 (47)	4,036 (56.1)	0.184	71 (68.3)	1,084 (63.8)	114 (61.3)	945 (58.5)	193 (53.5)	796 (55.2)	326 (56.3)	654 (53.5)	435 (46.3)	368 (42.6)	561 (38.7)	189 (53.8)	0.011

(DPP4i: dipeptidyl peptidase-4 inhibitor; SGLT2i: sodium-glucose co-transporter-2 inhibitor; GLP-1 RA: glucagon-like peptide-1 receptor agonist; ACEi: angiotensin-converting enzyme inhibitor; ARB: angiotensin receptor blocker; CCB: calcium channel blocker)

Table S4. Definitions of co-existing clinical disorders used as confounders for the Propensity Score Subclassification based on ICD (International Classification of Diseases, 10th version) and ICHI (International Classification of Health Intervention) codes.

Comorbidities: Definitions	
**Prior comorbidities**	**Description**
**Type 1 diabetes mellitus and type 2 diabetes mellitus**	Based on our diabetes definition.
**Atrial fibrillation**	Minimum 2 ICDs of atrial fibrillation (I48) together with the criteria of ICD repetition within a period more than 30 days but less or equal with 365 days (in- and outpatient records).
**Acut myocardial infarction with percutaneous coronary intervention**	One occurrence of defined ICDs in inpatient (primary or secondary diagnosis) records together with defined ICHIs within +- 30 days.<br>ICDs: I2100, I2110, I2120, I2130, I2140, I2190, I2200, I2210, I2280, I2290, I2300, I2310, I2320, I2330, I2340, I2350, I2360, I2370, I2380, I2390<br>ICHIs: 12730, 12731, 12740, 12750, 12751, 12752, 12754, 12760, 12780, 33970, 33971, 33972, 33974, 33975, 33976, 33981, 33982, 33983, 33984, 33985, 33986, 33987, 33988, 33989, 01339
**Coronary artery bypass surgery**	Occurrence of defined ICHIs in inpatient records.<br>ICHIs: 53611-17, 53621-23
**Cancer (as prior event)**	Minimum 2 ICDs of cancer (C) together with the criteria of ICD repetition within a period more than 30 days but less or equal with 365 days (in- and outpatient records).<br>ICDs: C00 - C99
**Heart failure**	Occurrence of HF ICDs in inpatient (primary diagnosis) records.<br>ICDs: I50, I42, I110, J81
**Chronic obstructive pulmonary disease**	Minimum 2 ICDs of COPD together with the criteria of ICD repetition within a period more than 30 days but less or equal with 365 days (in and outpatient records).<br>ICDs: J43, J44
**Keto-/lactate acidosis**	Occurrence of ICDs in inpatient (primary diagnosis) records.<br>E10.1, E11.1, E12.1, E13.1, E14.1, E87.2
**Lower limb amputations**	Occurrence of defined ICHIs in inpatient records.<br>ICHIs: 58450, 58460, 58470, 58471, 58480, 58530
**Major organ bleeding**	One occurrence of following bleeding ICDs in inpatient (primary diagnosis) records.<br>ICDs: D62, I60, I61, I62, R31, R04, D500, H313, H356, H431, H450, I312, I850, K250, K252, K254, K256, K260, K262, K264, K266, K270, K272, K274, K276, K280, K282, K284, K286, K625, K661, K920, K921, K922, S064, S065, S066, J942, K228F, K298A, K638B, K638C, K838F, K868G, I864A, H052A, S368D, G951A
**Lower extremity artery disease**	Occurrence of defined ICDs in inpatient (primary diagnosis) records.<br>ICDs: I701, I702, I703, I704, I705, I706, I707, I708, I709, I7039
**Percutaneous coronary intervention with stent**	Occurrence of defined ICHIs in inpatient records.<br>ICHIs: 33970, 33971, 33972, 33974, 33975, 33976, 33981, 33982, 33983, 33984, 33985, 33986, 33987, 33988, 33989, 01339
**Severe hypoglycemia**	Occurrence of ICDs in inpatient (primary diagnosis) records.<br>ICDs: E10.0, E11.0, E12.0, E13.0, E14.0, E16.0, E16.1, E16.2
**Stroke (as prior event)**	Occurrence of stroke ICDs in inpatient (primary diagnosis) records.<br>ICDs: I61, I6290, I63, I64
**Hypertension**	Minimum 2 ICDs of hypertension together with the criteria of ICD repetition within a period more than 30 days but less or equal with 365 days (in- and outpatient records).<br>ICD: I10H0
**Polycystic kidney disease**	Minimum 2 ICDs of PKD together with the criteria of ICD repetition within a period more than 30 days but less or equal with 365 days (in- and outpatient records).<br>ICDs: Q6110-Q6120-Q6130
**Glomerulonephritis**	Minimum 2 ICDs of glomerulonephritis together with the criteria of ICD repetition within a period more than 30 days but less or equal with 365 days (in- and outpatient records).<br>ICDs: N00-07
**Acute kidney injury**	Occurrence of AKI ICD in in- and outpatient records.<br>ICD: N17
**Septicemia**	Occurrence of septicemia ICD in in- and outpatient records.<br>ICD: A41
**Obesity**	Minimum 2 ICDs of obesity together with the criteria of ICD repetition within a period more than 30 days but less or equal with 365 days (in- and outpatient records).<br>ICD: E66

Table S5. List of accompanying medications used as confounders for Propensity Score Subclassification based on ATC (Anatomical Therapeutic Chemical) codes.

**Accompanying Medication: Definitions**	
**Drug**	**ATC Code**
**Blood glucose lowering drugs**	
Metformin	A10BA
Sulfonylureas	A10BB
DPP-4 inhibitors	A10BH, A10BD07, A10BD08, A10BD10, A10BD11
SGLT-2 inhibitors	A10BX09, A10BX11, A10BX12, A10BD15, A10BD16, A10BD20, A10BK
GLP1-RA	A10BX04, A10BX07, A10BX10, A10BX14, A10BJ
Metiglinides	A10BX02, A10BX03
Glitazones	A10BG, A10BD03, A10BD04, A10BD05
Acarbose	A10BF
Insulins	A10A
Short-acting	A10AB
Intermediate-acting	A10AC
Premixed	A10AD
Long-acting	A10AE
**Cardiovascular Disease Risk Treatment**	
Low dose acetylic salicylic acid	B01AC06
Statins	C10AA
Antihypertensives	
ACE inhibitors	C09A, C09B
ARB	C09C, C09D (exclude C09DX04)
Dihydropyridines (calcium channel blockers)	C08C
Low ceiling diuretics (thiazides)	C03A
Beta blockers	C07
Non-hydropyridines (calcium channel blockers)	C08D
High ceiling diuretics (loop-diuretics)	C03C
Aldosterone antagonists	C03DA
Digitoxin	C01AA04
Digoxin	C01AA05
Amiodarone	C01BD01
Warfarin	B01AA03
Receptor P2Y12 antagonists	B01AC04, B01AC22, B01AC24
Direct factor Xa inhibitors	B01AF
Direct thrombin inhibitor	B01AE07
Other antiplatelets	B01AC07, B01AC09, B01AC11, B01AC13, B01AC16, B01AC17, B01AC21, B01AC23
Corticosteroids	H02
Weight loss drugs	A08A
Antibiotics	J01

(DPP-4 inhibitors: dipeptidyl peptidase-4 inhibitors; SGLT-2 inhibitors: sodium-glucose co-transporter-2 inhibitors; GLP1-RA: glucagon-like peptide-1 receptor agonists; ACE inhibitors: angiotensin-converting enzyme inhibitors; ARB: angiotensin receptor blocker, CVD: cardiovascular disease)

Table S6. Definitions of clinical events used as primary endpoints based on ICD (International Classification of Diseases, 10th version) and ICHI (International Classification of Health Intervention) codes.

Primary endpoints	Description
**All-cause mortality**	Any kind of death, cause specific death is not available.
**Acut myocardial infarction with percutaneous coronary intervention**	One occurrence of defined ICDs in inpatient (primary or secondary diagnosis) records together with defined ICHIs within +- 30 days.<br>ICDs: I2100, I2110, I2120, I2130, I2140, I2190, I2200, I2210, I2280, I2290, I2300, I2310, I2320, I2330, I2340, I2350, I2360, I2370, I2380, I2390<br>ICHIs: 12730, 12731, 12740, 12750, 12751, 12752, 12754, 12760, 12780, 33970, 33971, 33972, 33974, 33975, 33976, 33981, 33982, 33983, 33984, 33985, 33986, 33987, 33988, 33989, 01339
**Stroke with CT/MR**	Occurrence of stroke ICD in inpatient (primary or secondary diagnosis) records together with defined CT/MRI ICHIs within +- 30 days.<br>stroke ICDs: I61, I6290, I63, I64<br>CT/MR ICHIs: 34410, 34411, 34412, 34490, 34914, 34915
**Hospitalization for heart failure**	Occurrence of HHF ICDs in inpatient (primary or secondary diagnosis) records.<br>ICDs: I50, I42, I110, J81
**Lower limb amputations**	Occurrence of defined ICHIs in inpatient records.<br>ICHIs: 58450, 58460, 58470, 58471, 58480, 58530
**Cancer**	Occurrence of cancer ICDs in inpatient (primary and secondary diagnosis) records.<br>ICDs: C00 - C99
**Kidney transplantation**	Occurrence of defined ICHIs in inpatient records.<br>ICHIs: 55551, 55553

Table S7. Prevalence of the 30 most frequent comorbidities observed in the CKD naïve group (left) and the CKD non-naïve group (right) in the preceding 5-years before the index date of the first dialysis.

CKD naïve (n=3,620)	Number of patients					
ICD-10	-5 year	-4 year	-3 year	-2 year	-1 year	total
Hypertension (I10) n, (%)	1,035 (28.6%)	1,090 (30.1%)	1130 (31.2%)	1,223 (33.8%)	2,212 (61.1%)	2,706 (74.8%)
Ischemic heart disease (I25) n, (%)	407 (11.2%)	438 (12.1%)	460 (12.7%)	488 (13.5%)	933 (25.8%)	1,242 (34.3%)
Anemia (D64) n, (%)	82 (2.3%)	90 (2.5%)	111 (3.1%)	170 (4.7%)	974 (26.9%)	1,171 (32.3%)
Non-insulin dependent diabetes mellitus (E11) n, (%)	485 (13.4%)	525 (14.5%)	551 (15.2%)	565 (15.6%)	856 (23.6%)	1,064 (29.4%)
Hospitalization for heart failure (I50) n, (%)	147 (4.1%)	164 (4.5%)	202 (5.6%)	229 (6.3%)	833 (23%)	978 (27%)
Lower extremity arterial disease (I70) n, (%)	221 (6.1%)	237 (6.5%)	260 (7.2%)	316 (8.7%)	620 (17.1%)	930 (25.7%)
Hyperlipidemia (E78) n, (%)	344 (9.5%)	351 (9.7%)	339 (9.4%)	352 (9.7%)	523 (14.4%)	902 (24.9%)
Abdominal pain (R10) n, (%)	137 (3.8%)	150 (4.1%)	156 (4.3%)	183 (5.1%)	517 (14.3%)	896 (24.8%)
Ametropia (H52) n, (%)	295 (8.1%)	291 (8%)	285 (7.9%)	267 (7.4%)	258 (7.1%)	829 (22.9%)
Spondylosis (M47) n, (%)	270 (7.5%)	297 (8.2%)	289 (8%)	273 (7.5%)	339 (9.4%)	807 (22.3%)
Pneumonia (J18) n, (%)	88 (2.4%)	95 (2.6%)	130 (3.6%)	128 (3.5%)	418 (11.5%)	715 (19.8%)
Cataract (H25) n, (%)	194 (5.4%)	210 (5.8%)	239 (6.6%)	247 (6.8%)	266 (7.3%)	667 (18.4%)
Angina pectoris (I20) n, (%)	249 (6.9%)	254 (7%)	260 (7.2%)	269 (7.4%)	429 (11.9%)	654 (18.1%)
Hyperplasia prostatae (N40) n, (%)	158 (4.4%)	180 (5%)	195 (5.4%)	213 (5.9%)	371 (10.2%)	583 (16.1%)
Atrial fibrillation (I48) n, (%)	154 (4.3%)	166 (4.6%)	195 (5.4%)	217 (6%)	476 (13.1%)	557 (15.4%)
Chronic obstructive pulmonary disease (J44) n, (%)	199 (5.5%)	205 (5.7%)	243 (6.7%)	250 (6.9%)	400 (11%)	551 (15.2%)
Gastrooesophageal reflux (K21) n, (%)	106 (2.9%)	119 (3.3%)	131 (3.6%)	148 (4.1%)	344 (9.5%)	551 (15.2%)
Retina disorders (H35) n, (%)	165 (4.6%)	176 (4.9%)	174 (4.8%)	205 (5.7%)	227 (6.3%)	505 (14%)
Intervertebral discus disorders (M51) n, (%)	170 (4.7%)	172 (4.8%)	193 (5.3%)	186 (5.1%)	207 (5.7%)	498 (13.8%)
Knee arthrosis (M17) n, (%)	177 (4.9%)	174 (4.8%)	181 (5%)	165 (4.6%)	190 (5.2%)	497 (13.7%)
Dorsalgia (M54) n, (%)	119 (3.3%)	138 (3.8%)	153 (4.2%)	144 (4%)	183 (5.1%)	489 (13.5%)
Obesity (E66) n, (%)	152 (4.2%)	151 (4.2%)	163 (4.5%)	162 (4.5%)	283 (7.8%)	469 (13%)
Fall (W19) n, (%)	107 (3%)	112 (3.1%)	110 (3%)	99 (2.7%)	161 (4.4%)	460 (12.7%)
Coxarthrosis (M16) n, (%)	150 (4.1%)	152 (4.2%)	163 (4.5%)	156 (4.3%)	194 (5.4%)	444 (12.3%)
Gastroduodenitis (K29) n, (%)	68 (1.9%)	69 (1.9%)	79 (2.2%)	94 (2.6%)	250 (6.9%)	438 (12.1%)
Electrolyte disorders (E87) n, (%)	21 (0.6%)	23 (0.6%)	40 (1.1%)	48 (1.3%)	340 (9.4%)	428 (11.8%)
Insulin-dependent diabetes mellitus (E10) n, (%)	211 (5.8%)	227 (6.3%)	247 (6.8%)	242 (6.7%)	316 (8.7%)	410 (11.3%)
Diabetes with complications (E14) n, (%)	157 (4.3%)	126 (3.5%)	144 (4%)	129 (3.6%)	203 (5.6%)	400 (11%)
Hyperuricemia (E79) n, (%)	57 (1.6%)	76 (2.1%)	78 (2.2%)	94 (2.6%)	278 (7.7%)	399 (11%)
Ear disorders (H61)n, (%)	119 (3.3%)	97 (2.7%)	108 (3%)	107 (3%)	112 (3.1%)	399 (11%)

CKD non-naïve (n=7,190)	Number of patients					
ICD-10	-5 year	-4 year	-3 year	-2 year	-1 year	total
Chronic kidney disease (N18) n, (%)	2,600 (36.2%)	3,251 (45.2%)	3,999 (55.6%)	4,942 (68.7%)	6,828 (95%)	7,116 (99%)
Hypertension (I10) n, (%)	4,049 (56.3%)	4,488 (62.4%)	4,813 (66.9%)	5,229 (72.7%)	6,478 (90.1%)	6,998 (97.3%)
Anemia (D64) n, (%)	628 (8.7%)	794 (11%)	1,020 (14.2%)	1,523 (21.2%)	3,358 (46.7%)	4,164 (57.9%)
Ischemic heart disease (I25) n, (%)	1,584 (22%)	1,759 (24.5%)	1,871 (26%)	2,093 (29.1%)	3,000 (41.7%)	3,859 (53.7%)
Non-insulin dependent diabetes mellitus (E11) n, (%)	2,189 (30.4%)	2,397 (33.3%)	2,532 (35.2%)	2,786 (38.7%)	3,238 (45%)	3,800 (52.9%)
Hyperlipidemia (E78) n, (%)	1,535 (21.3%)	1,669 (23.2%)	1,755 (24.4%)	1,999 (27.8%)	2,392 (33.3%)	3,595 (50%)
Lower extremity arterial disease (I70) n, (%)	875 (12.2%)	1,082 (15%)	1,230 (17.1%)	1,432 (19.9%)	2,201 (30.6%)	3,153 (43.9%)
Hospitalization for heart failure (I50) n, (%)	675 (9.4%)	762 (10.6%)	1,022 (14.2%)	1,274 (17.7%)	2,416 (33.6%)	3,055 (42.5%)
Hyperuricemia (E79) n, (%)	626 (8.7%)	763 (10.6%)	924 (12.9%)	1,126 (15.7%)	1,758 (24.5%)	2,501 (34.8%)
Ametropia (H52) n, (%)	889 (12.4%)	913 (12.7%)	886 (12.3%)	906 (12.6%)	956 (13.3%)	2,461 (34.2%)
Anemia other (D63) n, (%)	380 (5.3%)	490 (6.8%)	677 (9.4%)	1,002 (13.9%)	2,104 (29.3%)	2,412 (33.5%)
Insulin-dependent diabetes mellitus (E10) n, (%)	1,450 (20.2%)	1,533 (21.3%)	1,631 (22.7%)	1,731 (24.1%)	1,945 (27.1%)	2,378 (33.1%)
Angina pectoris (I20) n, (%)	839 (11.7%)	906 (12.6%)	948 (13.2%)	1,026 (14.3%)	1,245 (17.3%)	2,175 (30.3%)
Abdominal pain (R10) n, (%)	449 (6.2%)	501 (7%)	546 (7.6%)	547 (7.6%)	817 (11.4%)	2,087 (29%)
Spondylosis (M47) n, (%)	838 (11.7%)	804 (11.2%)	807 (11.2%)	812 (11.3%)	795 (11.1%)	2,079 (28.9%)
Retina disorders (H35) n, (%)	764 (10.6%)	819 (11.4%)	847 (11.8%)	911 (12.7%)	946 (13.2%)	2,036 (28.3%)
Cataract (H25) n, (%)	651 (9.1%)	716 (10%)	779 (10.8%)	848 (11.8%)	818 (11.4%)	1,990 (27.7%)
Pneumonia (J18) n, (%)	302 (4.2%)	366 (5.1%)	408 (5.7%)	467 (6.5%)	1,004 (14%)	1,964 (27.3%)
Gastrooesophageal reflux (K21) n, (%)	411 (5.7%)	471 (6.6%)	547 (7.6%)	644 (9%)	999 (13.9%)	1,720 (23.9%)
Diabetes with complications (E14) n, (%)	644 (9%)	663 (9.2%)	694 (9.7%)	710 (9.9%)	815 (11.3%)	1,718 (23.9%)
Obesity (E66) n, (%)	714 (9.9%)	741 (10.3%)	765 (10.6%)	806 (11.2%)	1,046 (14.5%)	1,662 (23.1%)
Electrolyte disorders (E87) n, (%)	151 (2.1%)	194 (2.7%)	287 (4%)	409 (5.7%)	1,104 (15.4%)	1,595 (22.2%)
Hyperplasia prostatae (N40) n, (%)	487 (6.8%)	592 (8.2%)	620 (8.6%)	705 (9.8%)	938 (13%)	1,533 (21.3%)
Atrial fibrillation (I48) n, (%)	444 (6.2%)	537 (7.5%)	651 (9.1%)	799 (11.1%)	1,247 (17.3%)	1,492 (20.8%)
Tubular renal disorders (N25) n, (%)	201 (2.8%)	304 (4.2%)	430 (6%)	702 (9.8%)	1,247 (17.3%)	1,436 (20%)
Urinal disorders (N39) n, (%)	305 (4.2%)	360 (5%)	391 (5.4%)	501 (7%)	804 (11.2%)	1,423 (19.8%)
Chronic obstructive pulmonary disease (J44) n, (%)	483 (6.7%)	541 (7.5%)	624 (8.7%)	735 (10.2%)	1,018 (14.2%)	1,413 (19.7%)
Chronic kidney disease other (N19) n, (%)	177 (2.5%)	235 (3.3%)	288 (4%)	414 (5.8%)	903 (12.6%)	1,399 (19.5%)
Gastroduodenitis (K29) n, (%)	247 (3.4%)	283 (3.9%)	357 (5%)	408 (5.7%)	727 (10.1%)	1,383 (19.2%)
Renal and uretral disorders (N28) n, (%)	311 (4.3%)	381 (5.3%)	400 (5.6%)	482 (6.7%)	693 (9.6%)	1,333 (18.5%)

The leading five comorbidities of the CKD naïve patients were hypertension, coronary artery disease, anemia, type 2 diabetes, and heart failure.

The leading five comorbidities of the CKD non-naïve patients was CKD, followed by hypertension, anemia, coronary artery disease, and type 2 diabetes.

Table S8. List of the 30 most redeemed medications in the CKD naïve patients in the preceding 5-years before the index date of the first dialysis.

	CKD naïve (n=3,620)	Number of patients					
ATC	ATC_NAME	-5 year	-4 year	-3 year	-2 year	-1 year	total
C03CA01	furosemide n, (%)	261 (7.2%)	310 (8.6%)	357 (9.9%)	435 (12%)	1,251 (34.6%)	1,557 (43%)
A12BA01	potassium chloride n, (%)	254 (7%)	288 (8%)	326 (9%)	410 (11.3%)	1,120 (30.9%)	1,484 (41%)
J01CR02	amoxicillin-clavulanic acid n, (%)	320 (8.8%)	332 (9.2%)	367 (10.1%)	395 (10.9%)	615 (17%)	1,379 (38.1%)
B01AC06	acetylsalicylic acid n, (%)	395 (10.9%)	413 (11.4%)	410 (11.3%)	382 (10.6%)	684 (18.9%)	1,240 (34.3%)
A02BC02	pantoprazole n, (%)	351 (9.7%)	368 (10.2%)	351 (9.7%)	406 (11.2%)	686 (19%)	1,180 (32.6%)
C08CA01	amlodipine n, (%)	366 (10.1%)	365 (10.1%)	389 (10.7%)	365 (10.1%)	661 (18.3%)	1,151 (31.8%)
J01MA02	ciprofloxacin n, (%)	192 (5.3%)	213 (5.9%)	242 (6.7%)	252 (7%)	467 (12.9%)	1,006 (27.8%)
M04AA01	allopurinol n, (%)	165 (4.6%)	220 (6.1%)	220 (6.1%)	260 (7.2%)	722 (19.9%)	990 (27.3%)
N02AX02	tramadol n, (%)	202 (5.6%)	233 (6.4%)	225 (6.2%)	286 (7.9%)	583 (16.1%)	978 (27%)
C10AA05	atorvastatin n, (%)	393 (10.9%)	356 (9.8%)	351 (9.7%)	338 (9.3%)	445 (12.3%)	936 (25.9%)
J01MA12	levofloxacin n, (%)	102 (2.8%)	147 (4.1%)	209 (5.8%)	250 (6.9%)	432 (11.9%)	839 (23.2%)
C09BA04	perindopril and diuretics n, (%)	165 (4.6%)	182 (5%)	228 (6.3%)	263 (7.3%)	579 (16%)	836 (23.1%)
B01AB05	enoxaparin n, (%)	115 (3.2%)	125 (3.5%)	157 (4.3%)	182 (5%)	426 (11.8%)	787 (21.7%)
C10AA07	rosuvastatin n, (%)	178 (4.9%)	222 (6.1%)	254 (7%)	326 (9%)	496 (13.7%)	780 (21.5%)
M03BX04	tolperisone n, (%)	200 (5.5%)	201 (5.6%)	196 (5.4%)	196 (5.4%)	269 (7.4%)	764 (21.1%)
C09AA04	perindopril n, (%)	191 (5.3%)	199 (5.5%)	195 (5.4%)	220 (6.1%)	424 (11.7%)	758 (20.9%)
A02BA03	famotidine n, (%)	189 (5.2%)	202 (5.6%)	159 (4.4%)	168 (4.6%)	434 (12%)	745 (20.6%)
M01AE02	naproxen n, (%)	98 (2.7%)	119 (3.3%)	154 (4.3%)	214 (5.9%)	354 (9.8%)	713 (19.7%)
C07AB12	nebivolol n, (%)	199 (5.5%)	189 (5.2%)	236 (6.5%)	242 (6.7%)	455 (12.6%)	702 (19.4%)
M01AB16	aceclofenac n, (%)	123 (3.4%)	132 (3.6%)	176 (4.9%)	177 (4.9%)	244 (6.7%)	646 (17.8%)
M01AB05	diclofenac n, (%)	218 (6%)	140 (3.9%)	133 (3.7%)	146 (4%)	249 (6.9%)	644 (17.8%)
A10BA02	metformin n, (%)	202 (5.6%)	232 (6.4%)	218 (6%)	229 (6.3%)	364 (10.1%)	638 (17.6%)
C03DA01	spironolactone n, (%)	93 (2.6%)	107 (3%)	140 (3.9%)	179 (4.9%)	521 (14.4%)	636 (17.6%)
C01DA02	glyceryl-trinitrate n, (%)	156 (4.3%)	162 (4.5%)	154 (4.3%)	164 (4.5%)	362 (10%)	585 (16.2%)
C09AA05	ramipril n, (%)	161 (4.4%)	168 (4.6%)	157 (4.3%)	185 (5.1%)	335 (9.3%)	567 (15.7%)
C07AB07	bisoprolol n, (%)	130 (3.6%)	145 (4%)	158 (4.4%)	197 (5.4%)	368 (10.2%)	548 (15.1%)
R03DA04	theophylline n, (%)	121 (3.3%)	127 (3.5%)	131 (3.6%)	164 (4.5%)	313 (8.6%)	544 (15%)
M01AX17	nimesulide n, (%)	125 (3.5%)	110 (3%)	121 (3.3%)	126 (3.5%)	203 (5.6%)	532 (14.7%)
C09BB04	perindopril and amlodipine n, (%)	66 (1.8%)	111 (3.1%)	136 (3.8%)	180 (5%)	319 (8.8%)	526 (14.5%)
J01FA10	azithromycin n, (%)	85 (2.3%)	121 (3.3%)	122 (3.4%)	138 (3.8%)	189 (5.2%)	521 (14.4%)

Table S9. List of the 30 most redeemed medications in the CKD non-naïve patients in the preceding 5-years before the index date of the first dialysis.

	CKD non-naïve (n=7,190)	Number of patients					
ATC	ATC Name	-5 year	-4 year	-3 year	-2 year	-1 year	total
C03CA01	furosemide n, (%)	1,288 (17.9%)	1,586 (22.1%)	1,812 (25.2%)	2,413 (33.6%)	4,337 (60.3%)	5,610 (78%)
M04AA01	allopurinol n, (%)	1,081 (15%)	1,242 (17.3%)	1,468 (20.4%)	1,745 (24.3%)	3,000 (41.7%)	4,617 (64.2%)
C08CA01	amlodipine n, (%)	1,570 (21.8%)	1,608 (22.4%)	1,691 (23.5%)	1,712 (23.8%)	2,340 (32.5%)	4,504 (62.6%)
A12BA01	potassium chloride n, (%)	972 (13.5%)	1,096 (15.2%)	1,244 (17.3%)	1,503 (20.9%)	2,370 (33%)	4,105 (57.1%)
J01CR02	amoxicillin-clavulanic acid n, (%)	1,076 (15%)	1,108 (15.4%)	1,158 (16.1%)	1,250 (17.4%)	1,584 (22%)	3,701 (51.5%)
B01AC06	acetylsalicylic acid n, (%)	1,336 (18.6%)	1,373 (19.1%)	1,371 (19.1%)	1,281 (17.8%)	1,692 (23.5%)	3,549 (49.4%)
B03XA01	erythropoietin (predialysis) n, (%)	571 (7.9%)	753 (10.5%)	1,029 (14.3%)	1,603 (22.3%)	3,375 (46.9%)	3,517 (48.9%)
A02BC02	pantoprazole n, (%)	1,082 (15%)	1,187 (16.5%)	1,365 (19%)	1,427 (19.8%)	1,789 (24.9%)	3,440 (47.8%)
C10AA05	atorvastatin n, (%)	1,306 (18.2%)	1,361 (18.9%)	1,355 (18.8%)	1,427 (19.8%)	1,725 (24%)	3,331 (46.3%)
B03AD03	iron-folic acid combination n, (%)	496 (6.9%)	596 (8.3%)	778 (10.8%)	1,101 (15.3%)	2,096 (29.2%)	3,161 (44%)
J01MA02	ciprofloxacin n, (%)	662 (9.2%)	736 (10.2%)	805 (11.2%)	838 (11.7%)	1,056 (14.7%)	2,760 (38.4%)
C02CA04	doxazosin n, (%)	675 (9.4%)	778 (10.8%)	892 (12.4%)	1151 (16%)	1,742 (24.2%)	2,745 (38.2%)
N02AX02	tramadol n, (%)	666 (9.3%)	743 (10.3%)	792 (11%)	909 (12.6%)	1,161 (16.1%)	2,486 (34.6%)
A11CC05	cholecalciferol n, (%)	326 (4.5%)	438 (6.1%)	629 (8.7%)	833 (11.6%)	1,500 (20.9%)	2,375 (33%)
C09BA04	perindopril and diuretics n, (%)	594 (8.3%)	722 (10%)	724 (10.1%)	846 (11.8%)	1,106 (15.4%)	2,260 (31.4%)
J01MA12	levofloxacin n, (%)	374 (5.2%)	464 (6.5%)	560 (7.8%)	716 (10%)	941 (13.1%)	2,209 (30.7%)
C07AB12	nebivolol n, (%)	641 (8.9%)	740 (10.3%)	810 (11.3%)	916 (12.7%)	1,276 (17.7%)	2,198 (30.6%)
C10AA07	rosuvastatin n, (%)	611 (8.5%)	784 (10.9%)	867 (12.1%)	986 (13.7%)	1,144 (15.9%)	2,172 (30.2%)
B01AB05	enoxaparin n, (%)	311 (4.3%)	368 (5.1%)	442 (6.1%)	601 (8.4%)	1,163 (16.2%)	2,150 (29.9%)
A02BA03	famotidine n, (%)	673 (9.4%)	616 (8.6%)	622 (8.7%)	639 (8.9%)	886 (12.3%)	2,002 (27.8%)
C09AA04	perindopril n, (%)	536 (7.5%)	548 (7.6%)	617 (8.6%)	661 (9.2%)	899 (12.5%)	1,986 (27.6%)
C01DA02	glyceryl-trinitrate n, (%)	532 (7.4%)	537 (7.5%)	565 (7.9%)	681 (9.5%)	1,001 (13.9%)	1,873 (26.1%)
C02AC06	rilmenidine n, (%)	297 (4.1%)	369 (5.1%)	494 (6.9%)	626 (8.7%)	1,089 (15.1%)	1,827 (25.4%)
C03DA01	spironolactone n, (%)	360 (5%)	408 (5.7%)	507 (7.1%)	597 (8.3%)	1,021 (14.2%)	1,780 (24.8%)
C07AG02	carvedilol n, (%)	662 (9.2%)	672 (9.3%)	686 (9.5%)	762 (10.6%)	1,065 (14.8%)	1,770 (24.6%)
M03BX04	tolperisone n, (%)	509 (7.1%)	520 (7.2%)	481 (6.7%)	457 (6.4%)	470 (6.5%)	1,717 (23.9%)
C03BA11	indapamide n, (%)	435 (6.1%)	522 (7.3%)	587 (8.2%)	587 (8.2%)	708 (9.8%)	1,704 (23.7%)
J01DC02	cefuroxime n, (%)	329 (4.6%)	375 (5.2%)	391 (5.4%)	456 (6.3%)	687 (9.6%)	1,703 (23.7%)
V06DD	amino acids n, (%)	86 (1.2%)	123 (1.7%)	194 (2.7%)	370 (5.1%)	1,415 (19.7%)	1,680 (23.4%)
C09AA05	ramipril n, (%)	553 (7.7%)	531 (7.4%)	554 (7.7%)	737 (10.3%)	591 (8.2%)	1,649 (22.9%)
