# Supplementary material for: Comparison of risks and costs of undiagnosed and diagnosed chronic kidney disease patients after the initiation of kidney replacement therapy in subsequent years. A Hungarian nationwide study
Source: Front Med (Lausanne). 2026 Jul 21;13:1759417. doi: 10.3389/fmed.2026.1759417 (PMC13434849; doi:10.3389/fmed.2026.1759417)
Supplement: Supplementary file 2 [file Data_Sheet_2.PDF]

Supplementary Material 2.

The original form of SQL script used for the patient selection to create the analytic cohorts of CKD naïve and CKD non-naïve patients from the Hungarian National Health Insurance Fund administrative database.
Translations and explanations of the Hungarian abbreviations and database fields are presented at the bottom of each page.

```
-- Transplanted patients
create table rxt_eskd_01_trans as
  select taj, trunc(beavdat, 'dd') datum
  from rxt_eskd_oep_efi
  where oenokod in ('55551', '55553') or tevkod in ('55551', '55553');

-- Dialysed patients
create table rxt_eskd_01_dial as
  select taj, kezdat datum, case when tevkod in ('88531', '88585', '88600', '88604') then 1 else 0 end
chronic
  from rxt_eskd_oep_muvese
  where tevkod like '8%' and tevkod <> '88586' and kezdat >= to_date('20090101', 'yyyymmdd');

-- Start and end of dialysis, presence of any chronic code
create table rxt_eskd_01_dial2 as
  select taj, min(datum) firstdate, max(datum) lastdate, max(chronic) chronic
  from rxt_eskd_01_dial
  group by taj;

-- Patients with dialyses for more than 30 days
create table rxt_eskd_01_dial3 as
  select a.taj, firstdate, lastdate, chronic
  from rxt_eskd_01_dial2 a
  where lastdate - firstdate > 30;

-- Patients with dialyses with chronic code or dying in 30 days after the first dialysis
-- Date of death (haldat) is known by month precision
create table rxt_eskd_01_dial4 as
  select a.taj, firstdate, lastdate, chronic
  from rxt_eskd_01_dial2 a
  inner join rxt_eskd_oep_taj b on a.taj = b.taj
  where chronic = 1 and to_date(substr(haldat, 1, 6), 'yyyymm') <= firstdate + 30;
```

---

oep – National Health Insurance Fund Administration
efi – case-based financing
TAJ – Hungarian Social Security Identification Number
datum – date of recorded medical event
beavdat – date of medical intervention
kezdat – start date of treatment
haldat – date of death
tevkod – medical procedure code
oenokod – Hungarian medical procedure coding system (OENO)
muvese – dialysis treatment
chronic – indicator of chronic kidney disease (1 = code present, 0 = otherwise)

```
-- Patients with dialyses with chronic code or dying in 30 days after the first dialysis
-- plus patients having a longer dialysis than 30 days
create table rxt_eskd_01_dial5 as
  select taj, firstdate, lastdate, chronic
  from rxt_eskd_01_dial3
union
  select taj, firstdate, lastdate, chronic
  from rxt_eskd_01_dial4;

-- The earlier of the first dialysis or transplantation, marking the type of the first treatment
-- This is the index date
create table rxt_eskd_01_indexdate as
  select distinct a.taj, a.datum, case when b.taj is not null then 'T' else 'D' end startreason
  from rxt_eskd_01_indexdate0 a
  left outer join rxt_eskd_01_trans b on a.taj = b.taj and a.datum = b.datum;

-- Any hospitalisation with 'N18' or 'N19' ICD codes not earlier than 5 years before the index date
-- (main and secondary diagnosis codes)
create table rxt_eskd_01_n18f as
  select a.taj, trunc(felvdat, 'dd') datum
  from rxt_eskd_oep_fekvo a
  inner join rxt_eskd_oep_fekvodiag b on a.kulcs = b.kulcs
  inner join rxt_eskd_01_indexdate k on a.taj = k.taj and trunc(felvdat, 'dd') + 5*365 >= k.datum
  where tipus in ('1', '2', '3', '4', '5') and substr(bno, 1, 3) in ('N18', 'N19');

-- Any outpatient visits with 'N18' or 'N19' ICD codes not earlier than 5 years before the index date
create table rxt_eskd_01_n18j as
  select a.taj, to_date(a.datum, 'yyyymmdd') datum
  from rxt_eskd_oep_jaro a
  inner join rxt_eskd_oep_jarodiag b on a.kulcs = b.kulcs
  inner join rxt_eskd_01_indexdate k on a.taj = k.taj and to_date(a.datum, 'yyyymmdd') + 5*365 >=
k.datum
  where substr(bno, 1, 3) in ('N18', 'N19');

-- The first 'N18' or 'N19' ICD code not earlier than 5 years before the index date (hospitalisation +
outpatient)
create table rxt_eskd_01_n18 as
  select taj, min(datum) datum
  from (select * from rxt_eskd_01_n18f union select * from rxt_eskd_01_n18j)
  group by taj
  having count(distinct datum) > 1;
```

---

oep – National Health Insurance Fund Administration
TAJ – Hungarian Social Security Identification Number
datum – date of recorded medical event
felvdat – hospital admission date
kulcs – unique record identifier used for linking database tables
tipus – diagnosis type identifier (main or secondary diagnosis)
bno – ICD10 diagnosis codes
startreason – type of first kidney replacement therapy (T = transplantation, D = dialysis)
fekvo – inpatient
jaro – outpatient

```
-- Any hospitalisation with 'N17' or 'A41' ICD codes in 60 days before the index date
-- (main and secondary diagnosis codes)
create table rxt_eskd_01_n17a41f as
  select a.taj, trunc(felvdat, 'dd') datum
  from rxt_eskd_oep_fekvo a
  inner join rxt_eskd_oep_fekvodiag b on a.kulcs = b.kulcs
  inner join rxt_eskd_01_indexdate k on a.taj = k.taj and trunc(felvdat, 'dd') between k.datum - 60 and
k.datum - 1
  where tipus in ('1', '2', '3', '4', '5') and substr(bno, 1, 3) in ('N17', 'A41');

-- Any outpatient visit with 'N17' or 'A41' ICD codes in 60 days before the index date
create table rxt_eskd_01_n17a41j as
  select a.taj, to_date(a.datum, 'yyyymmdd') datum
  from rxt_eskd_oep_jaro a
  inner join rxt_eskd_oep_jarodiag b on a.kulcs = b.kulcs
  inner join rxt_eskd_01_indexdate k on a.taj = k.taj and to_date(a.datum, 'yyyymmdd') between
k.datum - 60 and k.datum - 1
  where substr(bno, 1, 3) in ('N17', 'A41');

-- The last date with 'N17' or 'A41' ICD codes in 60 days before the index date (hospitalisation or
outpatient)
create table rxt_eskd_01_n17a41 as
  select taj, max(datum) datum
  from (select * from rxt_eskd_01_n17a41f union select * from rxt_eskd_01_n17a41j)
  group by taj;

-- EPO dates
create table rxt_eskd_01_eritropoetin as
  select distinct taj, trunc(kezdat, 'dd') datum
  from rxt_eskd_oep_muvese
  where tevkod = '59572' and kezdat >= to_date('20090101', 'yyyymmdd');

-- Excluded patients: Who had 'N17' or 'A41' ICD codes in 60 days before the index date
(hospitalisation or outpatient)
-- and had less than 8 EPO treatments before the last occurrence of the ICD codes above
create table rxt_eskd_01_exclude as
  select a.taj, count(distinct b.datum) darabszam
  from rxt_eskd_01_n17a41 a
  left outer join rxt_eskd_01_eritropoetin b on a.taj = b.taj and a.datum > b.datum
  group by a.taj
  having count(distinct b.datum) < 8;
```

---

oep – National Health Insurance Fund Administration
TAJ – Hungarian Social Security Identification Number
datum – date of recorded medical event
felvdat – hospital admission date
kulcs – unique record identifier used for linking database tables
bno – ICD10 diagnosis codes
kezdat – start date of treatment
tevkod – medical procedure code
muvese – dialysis treatment
darabszam – number of recorded treatment occurrences
fekvo – inpatient
jaro – outpatient

```
-- The earlier of these:
--   The first 'N18' or 'N19' ICD code not earlier than 5 years before the index date (hospitalisation + outpatient)
--   The first EPO treatment
create table rxt_eskd_01_naiv_e as
  select taj, min(datum) datum
  from (select * from rxt_eskd_01_n18 union select * from rxt_eskd_01_eritropoetin)
  group by taj;

-- Possible patients
create table rxt_eskd_patients as
  select a.taj, to_date(szuldat, 'yyyymmdd') dateofbirth, last_day(to_date(nvl(haldat, '29991231'), 'yyyymmdd')) dateofdeath,
    case when nem = '1' then 'male' else 'female' end sex, megye county, b.datum indexdate,
    case when e.taj is null then 'naive' else 'non naive' end naiv60,          -- naive if first N18, N19 or EPO is not more than 60 days before the indexdate
    case when trunc(MONTHS_BETWEEN (b.datum, to_date(szuldat, 'yyyymmdd') )/12)<=18 then '0-18'
      when trunc(MONTHS_BETWEEN (b.datum, to_date(szuldat, 'yyyymmdd') )/12)<=29 then '19-29'
      when trunc(MONTHS_BETWEEN (b.datum, to_date(szuldat, 'yyyymmdd') )/12)<=39 then '30-39'
      when trunc(MONTHS_BETWEEN (b.datum, to_date(szuldat, 'yyyymmdd') )/12)<=49 then '40-49'
      when trunc(MONTHS_BETWEEN (b.datum, to_date(szuldat, 'yyyymmdd') )/12)<=59 then '50-59'
      when trunc(MONTHS_BETWEEN (b.datum, to_date(szuldat, 'yyyymmdd') )/12)<=69 then '60-69'
      when trunc(MONTHS_BETWEEN (b.datum, to_date(szuldat, 'yyyymmdd') )/12)<=79 then '70-79'
      when trunc(MONTHS_BETWEEN (b.datum, to_date(szuldat, 'yyyymmdd') )/12)<=89 then '80-89'
    else '90-xx' end as agegroup,
    trunc(MONTHS_BETWEEN (b.datum, to_date(szuldat, 'yyyymmdd') )/12) age,
    startreason
  from rxt_eskd_oep_taj a
  inner join rxt_eskd_01_indexdate b on a.taj = b.taj
  left outer join rxt_eskd_01_naiv_e e on a.taj = e.taj and e.datum + 60 < b.datum
  left outer join rxt_eskd_01_exclude k on a.taj = k.taj
  where b.datum <= last_day(to_date(nvl(haldat, '29991231'), 'yyyymmdd'))      -- indexdate is before death
    and k.taj is null                                     -- not excluded
         and startreason = 'D';                           -- starts with dialysis, not transplantation

-- Included patients in PS matching
-- Index date between 2014 and 2018, age > 18
create table rxt_eskd_02_patients as
  select distinct taj, indexdate, sex, agegroup, to_char(indexdate, 'yyyy') indexyear, case when naiv60 = 'naive' then 1 else 0 end naive
  from rxt_eskd_patients
  where indexdate between to_date('20140101', 'yyyymmdd') and to_date('20181231', 'yyyymmdd') and agegroup <> '0-18';
```

---

oep - National Health Insurance Fund Administration
TAJ – Hungarian Social Security Identification Number
datum – date of the recorded medical event
szuldat – date of birth
haldat – date of death
megye – county of residence
indexdate – date of initiation of renal replacement therapy (dialysis or transplantation)
naiv60 – indicator of CKD naïve status (no CKD diagnosis or EPO treatment more than 60 days before dialysis initiation)
